# Classification of polyhedral shapes from individual anisotropically resolved cryo-electron tomography reconstructions

**DOI:** 10.1186/s12859-016-1107-5

**Published:** 2016-06-13

**Authors:** Sukantadev Bag, Michael B Prentice, Mingzhi Liang, Martin J Warren, Kingshuk Roy Choudhury

**Affiliations:** Statistics Department, University College Cork, Cork, Ireland; Department of Microbiology, University College Cork, Cork, Ireland; School of Biosciences, University of Kent, Canterbury, UK; Department of Biostatistics and Bioinformatics, Duke University, Durham, NC 27705 USA

**Keywords:** Polyhedron graph, Incomplete polyhedra, Classification from incomplete data, Cryo electron tomography, Bacterial microcompartment

## Abstract

**Background:**

Cryo-electron tomography (cryo-ET) enables 3D imaging of macromolecular structures. Reconstructed cryo-ET images have a “missing wedge” of data loss due to limitations in rotation of the mounting stage. Most current approaches for structure determination improve cryo-ET resolution either by some form of sub-tomogram averaging or template matching, respectively precluding detection of shapes that vary across objects or are a priori unknown. Various macromolecular structures possess polyhedral structure. We propose a classification method for polyhedral shapes from incomplete individual cryo-ET reconstructions, based on topological features of an extracted polyhedral graph (PG).

**Results:**

We outline a pipeline for extracting PG from 3-D cryo-ET reconstructions. For classification, we construct a reference library of regular polyhedra. Using geometric simulation, we construct a non-parametric estimate of the distribution of possible incomplete PGs. In studies with simulated data, a Bayes classifier constructed using these distributions has an average test set misclassification error of < 5 % with upto 30 % of the object missing, suggesting accurate polyhedral shape classification is possible from individual incomplete cryo-ET reconstructions. We also demonstrate how the method can be made robust to mis-specification of the PG using an SVM based classifier. The methodology is applied to cryo-ET reconstructions of 30 micro-compartments isolated from E. coli bacteria.

**Conclusions:**

The predicted shapes aren’t unique, but all belong to the non-symmetric Johnson solid family, illustrating the potential of this approach to study variation in polyhedral macromolecular structures.

**Electronic supplementary material:**

The online version of this article (doi:10.1186/s12859-016-1107-5) contains supplementary material, which is available to authorized users.

## Background

Cryo electron microscopy (cryo-EM) involves imaging biological samples flash frozen at cryogenic temperatures using a transmission electron microscope (TEM). Cryogenic freezing in a frozen-hydrated state prevents the biological sample from structurally deforming during sample preparation [1]. Unlike traditional TEM or X-ray crystallography, which also offer molecular to atomic level resolution, cryo-EM thus enables the imaging of macromolecular complexes, assemblies, cells and even tissues in a near native state [2].

**Fig. 1:**
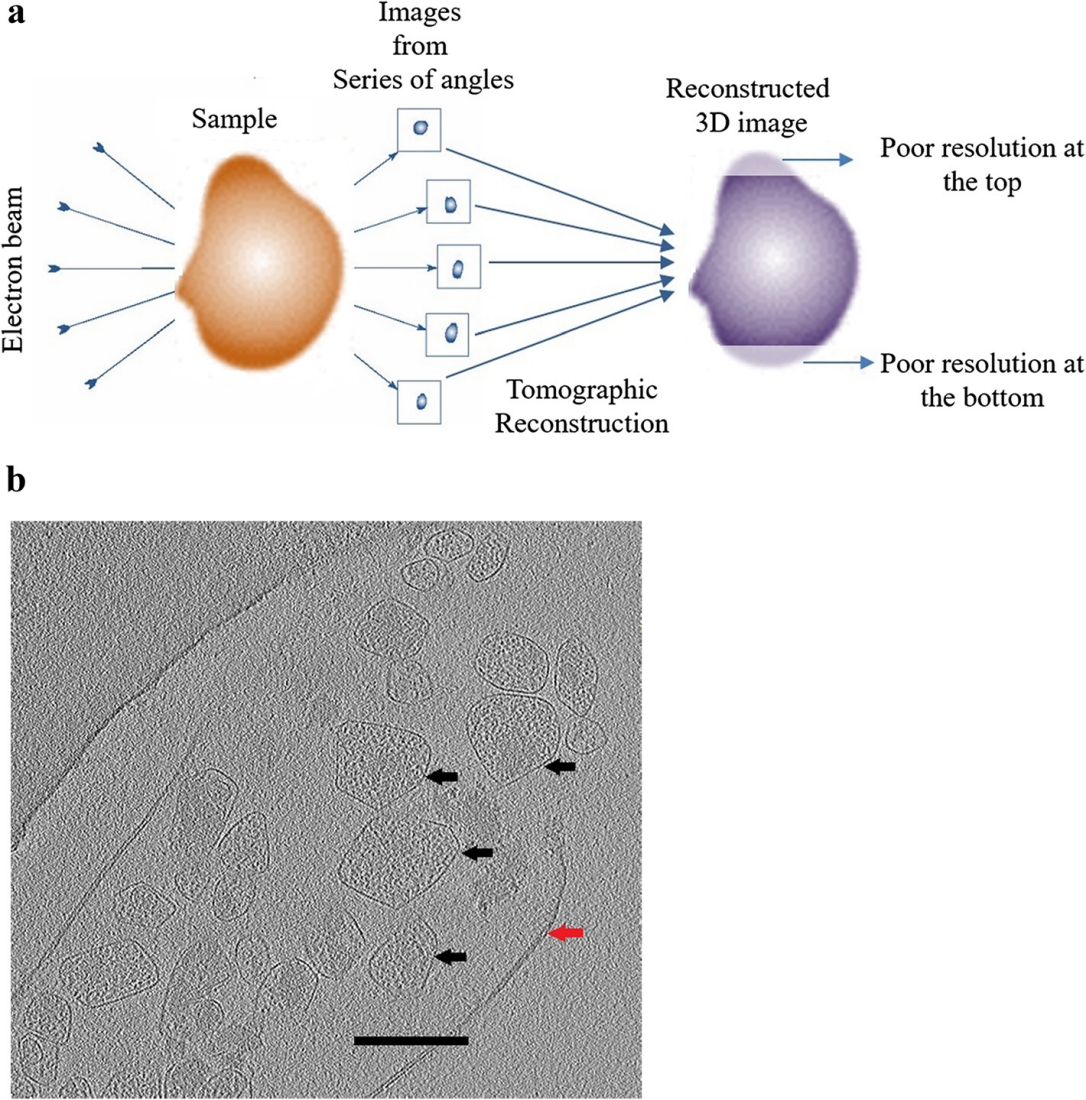
Cryo-EM tomography: **a** Schematic of single axis cryo-EM tomography. The (small) sample placed on a stage which is progressively tilted along a plane in a typical range of ± 60°, while exposed to an electron beam. Each tilt angle generates a planar projection image, which are collectively processed using an algorithm such as filtered back projection to generate a 3-d reconstruction of the original object. Due to the limited range of tilt angles, the reconstruction doesn’t have uniform resolution across the object. Typically, opposite ends (top and bottom) of the 3-d reconstruction in one direction have poor contrast resolution. **b** Mid-level cross-section from a 3-d cryo-EM reconstruction of an *E. Coli* cell. The small enclosures (black arrows) are micro-compartments (MC). The red arrow shows the cell membrane. Note that part of the cell membrane is missing. The scale bar is 200 nm

Cryo-electron tomography (cryo-ET) collects data by exposing the sample to an electron beam over multiple tilting angles (Fig. 1 a), enabling 3-D reconstruction of individual objects from the resulting 2-D projections. This reconstruction, which involves inversion of the 3-D Radon transform, does not require a priori assumptions about the objects structure [2]. However, there are a number of factors which limit the resolution of cryo-ET reconstructions. First, due to limitations in the degree of tilt of the mounting stage, the incomplete range of view angles causes a “missing wedge” in the Fourier (projection) domain data [2]. This in turn causes resolution of the 3-D reconstruction perpendicular to the sample surface to be worse than in the plane of the sample surface (Fig. 1b). Secondly, the total amount of radiation damage is proportional to the number of view angles times the radiation dose of the incident beam at a single view angle [2]. This multiplicative effect imposes strict constraints on intensity of the incident beam [3]. The consequently low and uneven resolution means that it can be difficult to identify the structure of individual objects from their cryo-ET reconstructions.

**Fig. 2:**
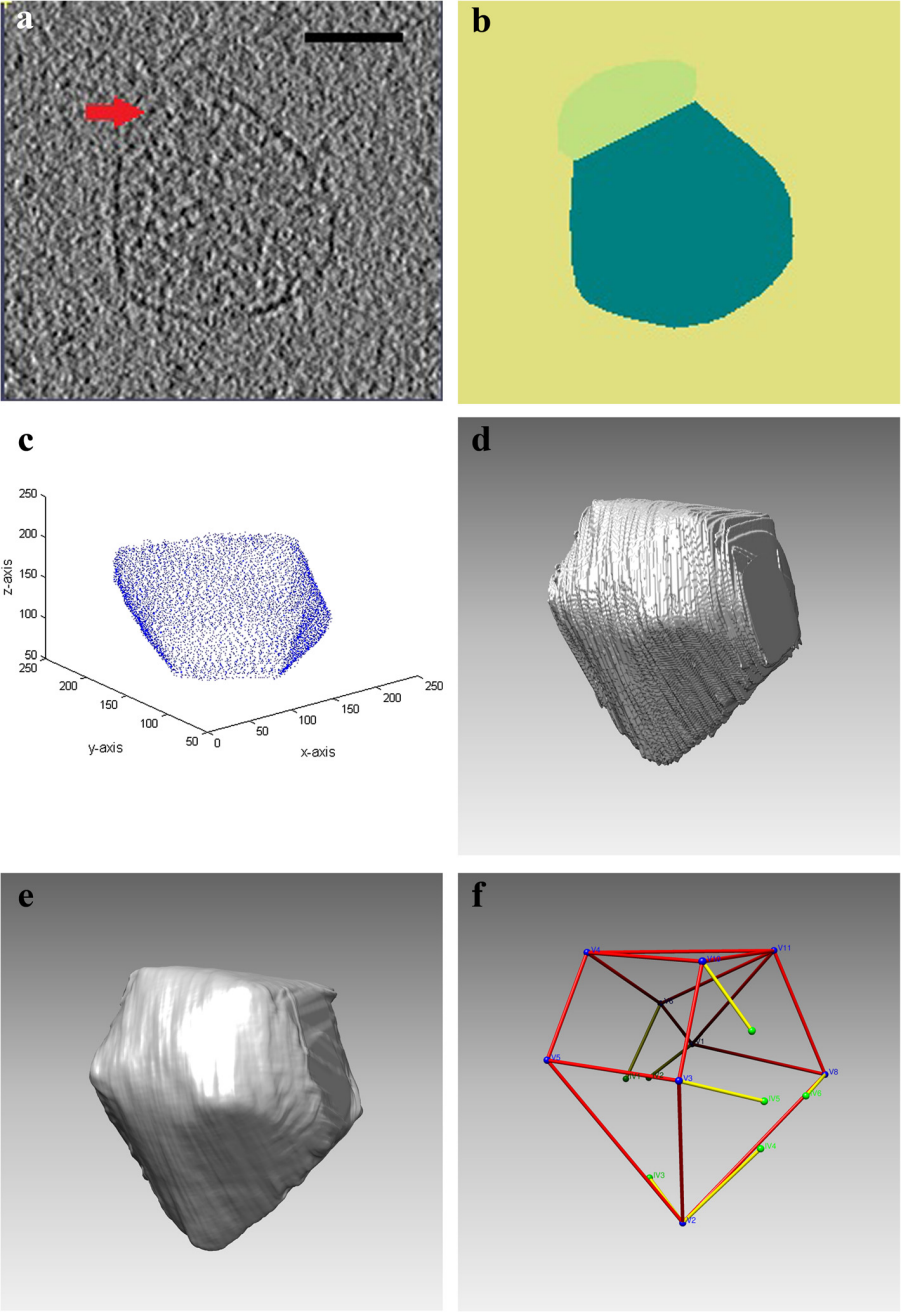
Extraction of polyhedral graph from cryo-EM reconstructions: **a** Upper level cross-sectional slice showing an MC with boundary partially visible due to poorer resolution. The scale bar is 50 nm. **b** Hand-drawn segmentation showing interior (deep green), exterior (yellow). Light green indicates a conservatively drawn region of uncertainty (caused by poor resolution) in which we suspect the object boundary lies. This region of uncertainty is used to constrain the 3-d reconstruction. **c** Stacked hand drawn boundaries from slices along the z-axis. Note that boundary information is completely missing for slices above and below this stack. **d** Volume rendering of regularized least squares reconstruction of object using data from stack in (**b**). Note missing wedge on right hand side. **e** Volume rendering of regularized least squares reconstruction of object using data from stacks of slices along x, y and z-axis. **f** Ball and stick diagram of polyhedral graph (PG) for object in (**e**), drawn using Chimera. Blue balls are observed vertices. Red lines are completed edges. Yellow lines are incomplete edges. Green balls are ends of incomplete edges

**Fig. 3:**
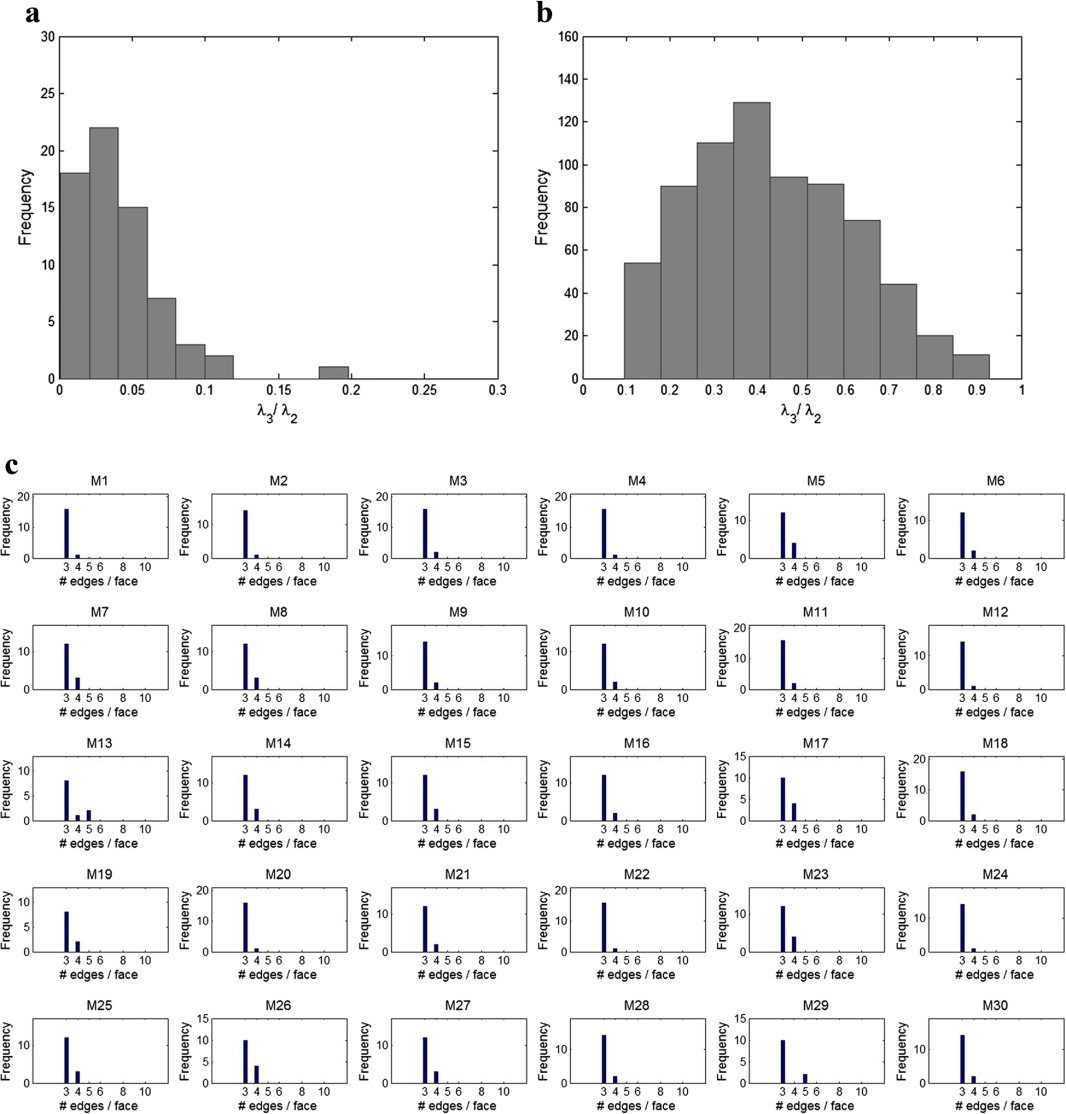
**a** Distribution of coplanarity test statistic (λ_3_/λ_2_) for observed non-triangular faces across 30 MCs. Note that λ_3_/λ_2_ = 0 if the ‘face’ is indeed planar. **b** Distribution of coplanarity test statistic obtained by combining pairs of adjacent triangular faces. Since adjacent faces of a convex polyhedron aren’t planar, we expect λ_3_/λ_2_ > 0. Based on these distributions, any identified ‘face’ with λ_3_/λ_2_ > 0.1 was declared non-planar and additional edges were added. **c** Distribution of observed edges for 30 MCs. The x-axis is no. of edges per face

Here we investigate the structure of bacterial micro-compartments (BMC): thin walled protein enclosures inside bacterial cells which separate certain metabolic pathways from the remaining cytoplasm [4]. Previous cryo-ET analyses of a class of BMCs known as carboxysomes, in two other strains of bacteria suggest that they have a polyhedral, specifically icosahedral, external structure [5, 6]. Visual inspection of reconstructed slices (Fig. 1b) and 3-d volume rendering (Fig. 2f) for our BMCs also suggest a convex polyhedral structure. In recombinant BMCs in *E.coli*, we demonstrate large variation in size and shape across copies within the same bacteria.

Previous methods identifying structure from cryo-ET reconstructions with missing wedge involves extracting multiple subvolumes (subtomograms) of the structure of interest, and then ‘averaging’ them, after appropriate alignment, to improve the resolution [7]. Another approach is by matching subtomograms against a high resolution template [8]. The limitations of present methods are thus: i) the structure of the template needs to be known/guessed in advance or ii) subtomogram averaging fails to capture variation of shapes across multiple copies of the object. Instead, we propose to realize the full potential of cryo-ET by identifying shapes using data from individual objects, without averaging of any sort or a priori assumptions about its shape. Further, we examine how accurately shapes can be identified from reconstructions of poor resolution using this methodology.

With a polyhedral structure in mind, we represent object shapes as incomplete polyhedral graphs (PG), i.e. a set of vertices connected by edges. We have developed a pipeline for extracting the PG from cryo-ET reconstructions. We also identify a library of reference polyhedra to which these objects should belong. Shape identification can thus be achieved by classifying the observed incomplete PG (which can be subject to measurement error) to one of the reference polyhedra. Apart from BMCs, these techniques could potentially also be applied to other biological objects exhibiting polyhedral structure, such as several types of viruses [1, 7, 9, 10] and protein complexes such as clathrin [11].

Developing optimal classification rules in this setting raises a number of methodological questions. Firstly, we need an appropriate stochastic model for PGs. Stochastic models for graphs typically assume that their edges are generated by a random process, e.g. Gaussian graphical models [12], exponentially generated random graphs [13] or stochastic block models [14]. In our library, two regular polyhedra can differ from each other in only a face or two. It would be complicated to define a stochastic model at edge level which could capture such small differences. Instead, we propose to model the observed PG as an incompletely sampled version of an underlying deterministic complete PG. Based on this model, we propose a method for estimating the sampling distribution of an incomplete PG for cryo-ET reconstructed images. Given that PGs are typically high dimensional, a general non-parametric density estimate would appear to suffer from the curse of dimensionality [15]. However, the highly structured form of PGs allows us to treat the sampling distribution like a discrete random variable with limited support, enabling convergence of the proposed density estimate at the parametric rate.

A second issue relates to how to incorporate information from edges that are only partially visible due to poor resolution (e.g. Fig. 2a): we can only identify one vertex of such an edge. Such edges cannot be incorporated into the adjacency matrix, which is commonly used to encode and analyse graphs [16]. To address this, we develop statistics for incomplete PGs, somewhat analogous to those used for right censored data.

Finally, previous approaches to classification with incomplete data involve a strategy of data augmentation, writing the posterior density as: *p*(*y*_*i*_|*x*_*i*_^*o*^) = ∫*p*(*y*_*i*_|*x*_*i*_^*o*^, *x*_*i*_^*m*^)*p*(*x*_*i*_^*m*^|*x*_*i*_^*o*^)*dx*_*i*_^*m*^, where *y*_*i*_ is the *i*-th class label, *x*_*i*_^*o*^ and *x*_*i*_^*m*^ are the observed and missing features respectively [17]. The difficulty in implementing this approach lies in constructing an appropriate model for *p*(*x*_*i*_^*m*^|*x*_*i*_^*o*^). Because the PG is uniquely specified by the polyhedron type, it is natural to first condition on *y*_*i*_, i.e. obtain *p*(*x*_*i*_^*m*^|*y*_*i*_, *x*_*i*_^*o*^) and then take an expectation over the polyhedron class, i.e. *p*(*x*_*i*_^*m*^|*x*_*i*_^*o*^) = ∑*p*(*x*_*i*_^*m*^|*y*_*i*_, *x*_*i*_^*o*^)*p*(*y*_*i*_). The marginal probability *p*(*x*_*i*_^*m*^|*x*_*i*_^*o*^) thus becomes dependent on the class of polyhedra chosen, making it a circular formulation. Instead, we propose a simpler procedure based solely on observed (incomplete) data. By modelling the incompleteness as a censoring mechanism, we propose a simulation based estimate of the probability density *p*(*x*_*i*_^*o*^|*y*_*i*_). We construct the Bayes classifier using this density estimate and demonstrate that this classifier is accurate for most polyhedra.

Extraction of the PG from tomographic reconstructions involves a number of processing steps such as vertex and edge identification, which are potentially liable to error, e.g. missing vertices and edges. We show the accuracy of the Bayes classifier seriously deteriorates in the presence of such errors. We propose two strategies for robust inference in this setting: i) selection of PG features, such as local topology, which are nearly preserved despite random missing edges or vertices ii) use of distance based classification methods, such as support vector machines (SVM), which can recognize near preservation. The methodology is illustrated by application to a set of *E. coli* MCs and the results are compared to those obtained for other types of bacteria.

## Methods

### Polyhedron models

We consider four families of convex polyhedral models, possessing varying degrees of symmetry, for the purposes of classifying data obtained from BMCs, namely the Platonic, Archimedean, Catalan and Johnson solids. Digital 3-d models of each of this library $\mathcal{P}$ of 123 polyhedra were constructed using a vertex enumeration algorithm [18]. The vertices and edges of each polyhedron were plotted using the UCSF Chimera (https://www.cgl.ucsf.edu/chimera/) to generate ‘ball-stick diagrams’, as shown in Fig. 6b. These ball stick diagrams were used to create meshes for each face of the polyhedron in MATLAB (www.mathworks.com). The meshes were combined to give a 3-d volume rendering of the polyhedron (see [19] for details).

## Extraction of the polyhedral graph (PG)

### Imaging and reconstruction

A single colony of *E.coli* cells expressing recombinant microcompartments was inoculated into NCE minimal medium supplemented with 1 % (w/v) succinate, 5 g/litre of yeast extract, 50 mM 1,2-propanediol, 20 μg/ml of tetracycline, 30 μg/ml of cefsulodin. Cells were grown at 37 °C with shaking for 24 h. The culture OD600 was adjusted to 0.5 with NCE minimal medium, mixed with 10 nm colloidal gold and applied to holey carbon grids without any centrifugation. Excess solution was blotted away with filter paper in a 100 % relative humidity chamber and grids were then plunge-frozen in liquid ethane and propane mixture (37:67) with a Vitrobot (FEI, Netherlands). The sample was imaged using single-axis tilt angle cryo-ET at 300 kV on FEI G2 Polara transmission electron microscope. Images were collected on a lens coupled 4 k x 4 k UltraCam (GATAN, Pleasanton, CA).

During imaging, the sample tilts around a single axis from -60° to +60° with 1° intervals, yielding projection images at each orientation (Fig. 1a). These 121 projections were aligned and digitally reconstructed by inverting a Radon transform with cone beam geometry using least squares based filtered backprojection in the IMOD etomo module [20]. The reconstructed 3-d volumes have multiple BMCs in the field of view (Additional file 5: Figure S11), which were isolated as subtomograms using the IMOD trimvol module (Fig. 1b). Our goal is to identify the vertices and edges on each of these reconstructed BMCs and from this, construct their PG. Our identification algorithm has two steps: i) Obtain ‘cleaned’ 3-d volume renderings for each of the objects in the field of view. ii) Identify edges and vertices from the cleaned volume renderings.

### Segmentation and volume rendering

Ideally, we would like to apply an automated segmentation algorithm to objectively isolate the 3-d volume of each BMC. But BMC boundaries in the 3-d reconstructed volumes are sometimes indistinct and they also possess internal texture (Fig. 2a), meaning that automated segmentation using standard approaches such as edge detection, seeded region growing etc. [13] yield poor results. To suppress texture and maximize edge contrast, we adopted a two step approach to segmentation: *a*) extraction of slicewise object boundaries in different orientations; *b*) reconstruction of object surface from the collection of object boundaries. For step *a*), the 3-d volume was re-sliced in 3 orthogonal directions (approximately 100 slices each in the *x, y* and *z* directions) using Amira (www.amira.com). Object boundaries were marked by manual tracing on each slice using MATLAB (Fig. 2b), yielding a point-cloud of the surface (Fig. 2c). Although this exercise is quite tedious and time consuming (tracing for each object took about 6 h), we undertook re-slicing in orthogonal directions because the optimal direction for edge-contrast can vary depending on the 3-d orientation of the normal vector at any given point on the surface.

In step b), for each set of *x, y* and *z* slices, we define directional profiles *f*_*x*_(*x,y,z*), *f*_*y*_(*x,y,z*) and *f*_*z*_(*x,y,z*). On a given slice, the interior profile is defined to be = 1 for points inside the convex hull of the boundary points (Fig. 2b). When the object boundary is closed, the exterior profile is defined to be = 0 for points outside the convex hull of the boundary points (the exterior). When the object boundary isn’t closed (i.e. not completely visible), the exterior is obtained by extending either end of the visible boundary intersected by a bounding box (Fig. 2b). Points which are neither in the interior or the exterior on the slice are defined as missing (Fig. 2b). To reconstruct the object, *f*(*x,y,z*), we use the least squares fitting criterion *L*(*x*, *y*, *z*) = ((*f*(*x*, *y*, *z*) − *f*_*x*_(*x*, *y*, *z*))^2^ + (*f*(*x*, *y*, *z*) − *f*_*y*_(*x*, *y*, *z*))^2^ + (*f*(*x*, *y*, *z*) − *f*_*z*_(*x*, *y*, *z*))^2^) which yields the pointwise mean of *f*_*x*_, *f*_*y*_ and *f*_*z*_ as the least squares estimate of *f*. To account for missing data, the least squares estimate is modified to be the mean of all non-missing *f*_*x*_, *f*_*y*_ and *f*_*z*_ values (Fig. 2d). The estimate is then post processed using a Gaussian filter (Fig. 2e).

### Measuring size and shape of reconstructed micro-compartments

Volumes of the reconstructed BMCs were measured by counting voxels in their interior, i.e. *n* = #*W*, where *W* is a matrix whose rows are 3-d points {(*x,y,z*) : *f*(*x,y,z*) > 0.5}. The gross shape of the BMCs was studied by fitting an ellipsoidal model. Their sphericity was assessed using *sp*_2_ = *r*_2_/*r*_1_ and *sp*_3_ = *r*_3_/*r*_1_, where *r*_3_ ≤ *r*_2_ ≤ *r*_1_, are the lengths of the principal axes of an ellipsoid, obtained as the eigenvalues of *W*^*T*^*W,* obtained from least squares fitting of an ellipsoid to the point set defined by the rows of *W* [21]. For a spherical object, we expect *sp*_2_ = *sp*_3_ = 1. Given this variability, in this paper we focus on the combinatorial structure of a polyhedron, which is invariant with respect to invertible affine transformations, such as changes in scale, rotation, translation, shear, similarity etc. In particular, straight lines and planes, which form the edges and faces of a polyhedron, are preserved under invariant affine transformations [22].

### Identifying vertices and faces

Our algorithm for identification of vertices and faces on the object involves two steps. i) Visual identification of potential vertices and edges ii) Validation. Identification and labelling was done in the UCSF Chimera software [23], which allows for convenient 3-d visualization and annotation of edges and vertices. The annotation distinguishes between complete and incomplete edges (Fig. 2f). For validation, we exploit the fact that a polyhedron is composed of planar faces. Given a set of vertices *V*_1_, *V*_2_,…, *V*_*k*_ that purport to form a face, i.e. edges between these vertices form a cyclic graph, we compute the 3x 3 matrix *VV*^*T*^ where *V* = (*V*_1_, *V*_2_,…, *V*_*k*_) is a 3 x *k* matrix comprising co-ordinates of the vertices. Next we compute the eigen-values *λ*_1_ ≥ *λ*_2_ ≥ *λ*_3_ of *VV*^*T*^*.* If the points are exactly co-planar, we expect *λ*_3_ = 0. To test the hypothesis H_0_: Vertices are co-planar vs. H_1_: Vertices are not co-planar, we compute distribution of the test statistic $T={\widehat{\lambda}}_3/{\widehat{\lambda}}_2.$ The null and alternate distribution of the test statistic are computed respectively from i) visually identified quadrilateral faces and ii) four vertex sets comprising adjacent triangular faces (Fig. 3a). Using the rule to reject H_0_ if *T* > 0.1, we identified 68 quadrilateral or pentagonal faces and 717 triangular faces in the 30 BMCs. Based on this identification, the 30 BMCs had between 10 and 18 faces each, with the median being 15 (Additional file 1: Table S4). Most BMCs had 1-2 quadrilateral faces, with the rest being triangular faces (Fig. 3c). The non-degenerate face type distribution suggests that a Platonic model may not be appropriate for BMCs.

### Regularity of faces

We measure the regularity of an identified face using *r*_*e*_ = *s*_*e*_/*ē*, where *ē* and *s*_*e*_ are the sample mean and standard deviation of all edge lengths in the face. This is a dimensionless measure: if a face is a regular polygon, we expect *r*_*e*_ = 0. For an isosceles triangle with edge lengths 1, 2 and 2, *r*_*e*_ = 0.34. The *r*_*eij*_ statistic was analyzed across identified faces *j* within BMCs *i* =1,…, 30, using the model *r*_*eij*_ = *μ* + *m*_*j*_ + *ε*_*ij*_, where μ is the overall mean regularity, *m*_*j*_ is the effect of BMC and ε_*ij*_ is the within BMC variability, both assumed to have independent random Gaussian distributions with mean 0 and SD σ_*m*_ and σ_*f*_ respectively. This analysis yielded $\widehat{\mu}=0.22$, ${\widehat{\sigma}}_f=0.10$ and ${\widehat{\sigma}}_m=0.03$, suggesting that i) faces are non-regular polygons ii) the degree of non-regularity varies within an object. Both these conclusions point to the difficulty of using metric properties, i.e. lengths, distances and angles of the BMCs to predict their structure with a polyhedron model. Consequently, we focus solely on their topological properties.

### Topological features of a polyhedron graph

Steinitz’s theorem states that any (3D) convex polyhedron is isomorphic to a planar graph, known as the polyhedral graph (PG) [22]. The structure of a graph is captured by its adjacency matrix *A* = ((*a*_*ij*_)), where *a*_*ij*_ = 1 if there is an edge between the *i*-th and *j*-th vertices, 0 otherwise. Important *global* properties of a graph include the number of vertices *V*, edges, *E* and faces *F* [16]. For incomplete polyhedra, we note that each of these measures is right censored. Further, because edges could be counted when vertices are missing, Euler’s well known relation for convex polyhedra, *V* - *E* + *F* = 2 does not hold for incomplete data.

We also consider local topological properties *L* such as the distribution of face types (*F*_3_, *F*_4_, *F*_5_, *F*_6_) i.e. count of the number of triangles, quadrilaterals, pentagons and hexagons in each BMC (Fig. 3c) and the distribution of vertex degree (*V*_3_, *V*_4_, *V*_5_, *V*_6_), i.e. the number of edges connected to a vertex (Additional file 2: Figure S7). We also consider higher order topological properties, namely i) edge adjacency matrix, *EV*, where *EV*_*ij*_ = # edges with vertices of degree *i* and *j* at either end and ii) face adjacency matrix, *FV*, where *FV*_*ij*_ = # edges with faces comprising *i* and *j* vertices on either side. These local topological features can be informative about the choice of polyhedral model from incomplete observations: we have already noted how the non-degenerate face type distributions can help rule out Platonic solids. Similarly, the non-degenerate vertex degree distribution of BMCs helps rule out Archimedean solids (Additional file 2: Figure S3 and S7).

Local topological properties are recorded in two versions: *complete* and *incomplete*. The complete version is based on only those features which are completely observed, e.g. a closed triangular face with all three edges completely visible. A limitation of the adjacency matrix is that it cannot accommodate edges where one vertex is missing (Fig. 2f). To capture this information, we propose extensions of topological features for incomplete data, e.g. a face with three edges visible and one side open (Fig. 2f): it could be a face with 3, 4 or 5 (or more) edges. To reflect this ambiguity, we create a cumulative right censored version of the face type distribution: (*F*_3+_, *F*_4+_, *F*_5+_, *F*_6+_), where *F*_3+_ is the number of faces with at least 3 edges, etc. An analogous distribution (*V*_3+_, *V*_4+_, *V*_5+_, *V*_6+_), is created for vertex degree. The collection of all these features is termed the topological profile (TP) of a polyhedron (Table 1). The list of all features for the 123 solids in $\mathcal{P}$ is shown in Additional file 3: Table S3 and for the 30 BMCs in Additional file 1: Table S4.

**Table 1:** Categorization of features in the topological profile (TP) of a polyhedral graph (PG)

		Feature type			
Topological profile component	Dimension	Complete	Incomplete	Global	Local
V,E,F	3	x		x	
Face type distribution	6	x			x
Vertex degree distribution	6	x			x
At least face type distribution	8		x		x
At least vertex type distribution	8		x		x
Edge adjacency matrix	10 × 10 = 100	x			x
Face adjacency matrix	10 × 10 = 100	x			x
Total	231				

## Characterizing distribution of truncated polyhedra

**Fig. 4:**
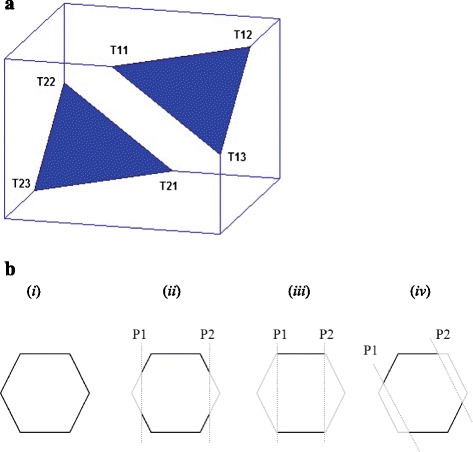
Truncation of polyhedra to simulate the missing wedge and its effect on the polyhedral graph (PG): **a** A cube (Platonic polyhedron originally with *V* = 8, *E* = 12, *F* = 6) truncated by two parallel planes, resulting in *V* = 6, *E* = 6, *F* = 0 (completely observed), *F*_3_ + = *F*_4_ + = 6 (incompletely observed faces). **b** Effect of varying the orientation of the truncation planes. (*i*) Original hexagon *V* = 6, *E* = 6, *V*_2_ = 6 (*ii*) *V* = 4, *E* = 2, *V*_2_ = 4 (*iii*) *V* = 4, *E* = 2, *V*_1_ = 4 (*iv*) *V* = 2, *E* = 2, *V*_2_ = 2

In order to compare the PG of a BMC to that of a polyhedral model, we need to account for the effect of missing sections in a polyhedral model on its PG. As seen in Figs. 1a and 2d, the effect of the missing sections can be approximated by slicing off two end sections of the polyhedron by parallel planes (Fig. 4a). The structure of the resulting PG, as well as features derived from it, e.g. the number of vertices, edges etc. as well as face types, depends on *i*) the orientation *ϕ* of the plane(s) *ii*) the perpendicular distance *d* of the plane from the centroid of the polyhedron *μ* (Fig. 4b). Since truncation is a geometric operation, we operate on the matrices formed by the Cartesian co-ordinates of the vertices: *V*_P_ = (*V*_1_,..,*V*_m_), a 3 x *m* matrix and discretized edges of a polyhedron model *E*_P_ = (*E*_1_,..,*E*_*le*_), a 3 x *le* matrix, where *e* is the number of edges and each edge i.e. line segment is discretized into a set of *l* equispaced points in 3-d. Any pair of truncating planes can be obtained by specifying two quantities: *a*) the perpendicular distance *d* between the plane and the centroid of vertices of the PG, *μ*_*V*_ = *m*^− 1^∑_*i* = 1_^*m*^*V*_*i*_*.* The distance *d* represents the amount of truncation which is determined by the imaging protocol and the size of the object. The normalized truncation percentage is obtained as 200*(1 – *d/d*_max_), where *d*_max_ = max ||*V*_*i*_ – μ_*V*_||, *i* = 1,…., *m. b)* the random orientation *ϕ* of the normal to the truncation plane, which is obtained by sampling *ϕ* = (*ϕ*_x_, *ϕ*_y_) ~ U[0, 2π] x U[0, 2π]. The vertices and edges of the rotated polyhedron are obtained as *RV*_P_ = *R*_*ϕ*_*V*_P_ and *RE*_P_ = *R*_*ϕ*_*E*_P_, where *R*_*ϕ*_ is the 3 x 3 rotation operator matrix with rotation angle *ϕ*. The vertices and edges of the truncated and rotated polyhedron are obtained by excluding all vertices *TRV*_*P*_ = {*RV*_*i*_: ||*RV*_*i*_ – μ_*V*_|| > *d*, *i* = 1,…., *n*} and all portions of discretized edges *TRE*_*P*_ = {*RE*_*iP*_: ||*RE*_*ki*_ – μ_*V*_|| > *d, k =* 1*,…,l, i =* 1*,…,e*}*.*

**Fig. 5:**
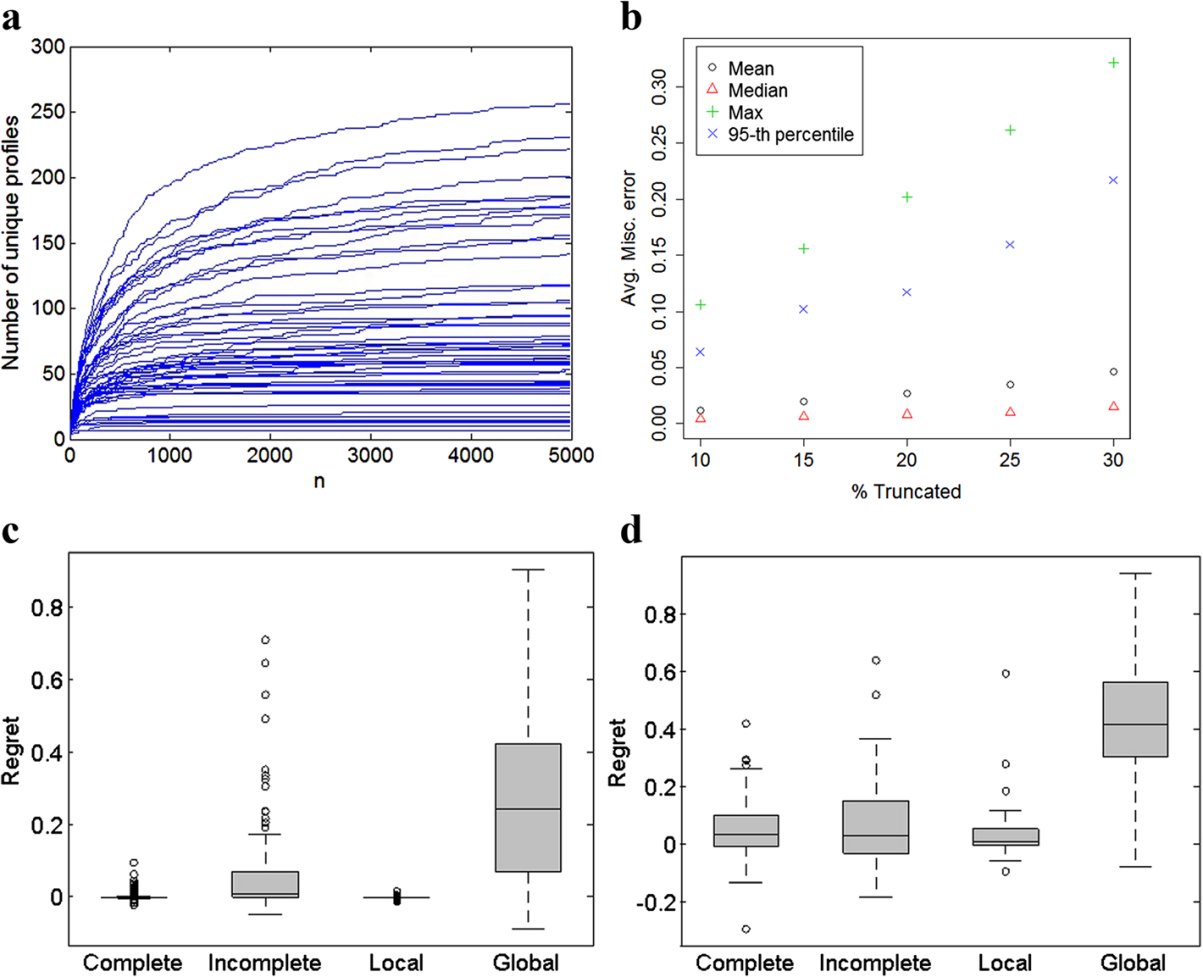
**a** Estimation of the minimum size of training set required for classification of truncated polyhedra. Each line in the graph represents a polyhedron from the family $\mathcal{P}$. Only polyhedra with ≤ 20 vertices are shown (55 such). Each solid is randomly rotated *n* times and truncated by parallel planes to achieve 20 % truncation. For each truncated solid so obtained, a truncated topological profile (*TTP*) is created. Due to internal symmetries in the polyhedron, many of these topological profiles are identical. The y-axis counts the number of unique topological profiles obtained from these *n* truncated solids. **b** Misclassification error of the Bayes classifier as a function of the percent polyhedron truncated. The misclassification error was calculated using a test set of 500 randomly selected TTPs from each of 115 solids with unique complete topological profiles. **c** Comparison of topological feature subset specific misclassification errors for the Bayes classifier (115 solids). Regret is defined as the difference in misclassification errors achieved when classifying using just the features in the named subset versus using all features. See Table 1 for definition of subsets. **d** Comparison of topological feature subset specific misclassification errors for the SVM classifier (54 solids). See Table 1 for definition of subsets. Note that the test set polyhedral graphs is mis-specified for this calculation

The PG of the truncated polyhedron, constructed from *TRV*_*P*_ and *TRE*_*P*_. Its topological profile is denoted as a truncated topological profile (*TTP*). To determine how many orientations *n* need to be sampled to ensure adequate coverage of all possible truncated polyhedra arising from a given polyhedron $\varTheta \in \mathcal{P}$, we examined the dependence of number of unique *TTP*s on *n*. As there were very few or no new unique *TTP*s being generated beyond *n* = 4500 sampled orientation for most polyhedra (Fig. 5a), we decided this was adequate. The relatively small number of unique profiles also indicates that probability distribution of *TTP*s, *T,* for a given polyhedron $\varTheta \in \mathcal{P}$, *p*(*T*|*Θ*) has limited finite support. It follows that *p*(*T*|*Θ*) can be estimated by the empirical discrete density function: $\widehat{p}\left(T\Big|\varTheta \right)={n}^{-1}{\displaystyle \sum_{i=1}^nI\left(T{P}_i=T\right)}$, where *I*() is the indicator function. Using the properties of the Bernoulli distribution, we note that $\widehat{p}\left(T\Big|\varTheta \right)$ is an unbiased estimate of the density *p*(*T |* Θ**)** and converges to it at the usual parametric rate, with variance *n*^− 1^*p*(*T*|*Θ*) (1 − *p*(*T*|*Θ*)). The finite support suggests that the *TTP* are a form of indexing or *hashing* of the underlying truncated polyhedra.

### Bayes classifier

Given the estimated TTP distributions $\widehat{p}\left(T\Big|\varTheta \right)$ for all polyhedra $\varTheta \in \mathcal{P}$, the Bayes classifier can be estimated as $\widehat{\varTheta}(T)= \arg \max \widehat{p}\left(\left.\varTheta \right|T\right)$, where the posterior density is obtained as:

1 $$ \widehat{p}\left(\left.\varTheta \right|T\right)=\frac{\widehat{p}\left(\left.T\right|\varTheta \right)p\left(\varTheta \right)}{{\displaystyle {\sum}_{\theta \in \mathcal{P}}\widehat{p}\left(\left.T\right|\theta \right)p\left(\theta \right)}} $$

The accuracy of the classification rule is evaluated by generating an independent set of truncated test profiles *T*_*i*_^*Θ*^, *i* = 1,2,…, *nt* for all polyhedra $$ \varTheta \in \mathcal{P} $$. The rank of the matrix of training set features TP (effective dimension) was 44. We identified 8 pairs of solids in $$ \mathcal{P} $$ which share the exact same (complete) polyhedral profile (Additional file 3: Table S3). For identifiability, we will identify these pairs as the same solid during classification, leaving us with 115 solids. We assume a non-informative uniform prior over these 115 solids. Misclassification occurs if the classification rule predicts the wrong structure based on the observed polyhedral graph of the object. The overall misclassification error is defined as $$ m\left(\varTheta \right)={\displaystyle {\sum}_{i=1}^{nt}I\left(\widehat{\varTheta}\left({T}_i^{\varTheta}\right)\ne \varTheta \right)} $$ and confusion matrix $$ {M}_{\varTheta \hbox{'},\varTheta }={\displaystyle {\sum}_{i=1}^{nt}I\left(\widehat{\varTheta}\left({T}_i^{\varTheta}\right)=\varTheta \hbox{'}\right)} $$$$ \forall \kern0.5em \varTheta \hbox{'},\kern0.5em \varTheta \in \mathcal{P} $$. Since simulated data are generated from a known polyhedral model, it is possible to estimate average misclassification errors in this setting by generating many replicates datasets from the same polyhedron model.

## Results

### Features of polyhedral models

At the highest level of symmetry are the 5 platonic solids, which have all faces congruent and an equal number of edges meet at each vertex. Thus both the distribution of number of edges per face and number of edges per vertex (vertex degree) is degenerate for these solids (Additional file 2: Figure S1 and S2). All 5 platonic solids have been used as models for molecular and crystal structure. In particular, the icosahedron has been identified as the structure for BMCs occurring in two other strains of bacteria [5, 6]. At the next level of symmetry are the 13 Archimedean solids, which have a unique vertex degree, but two types of faces (Additional file 2: Figure S3 and S4). Nine of the Archimedean solids can be obtained by truncation of Platonic solids, so they are plausible candidates, given the truncated nature of the observed data. Their counterparts are 13 Catalan solids, which are duals of the Archimedean solids, in the sense that they have a unique face type, but two types of vertex degree. At the lowest level of symmetry are the Johnson solids, which have regular polygons as faces, but there is no restriction on which faces can appear on a polyhedron. It was proved that there are only 92 such convex polyhedra possible [24]. The distribution of both the vertex degree and face type is non-degenerate for Johnson solids (Additional file 2: Figure S5 and S6).

### Size and shape of reconstructed micro-compartments

Objects whose largest cross-sectional slices were found too small to visually trace edges were were excluded due to difficulty in segmentation. The remaining 30 segmented BMCs range in volume from 0.04 to 3.35 attolitres (=10^-18^ litre), with a mean of 1.15 attolitres. In the 30 BMCs, mean (SD) of *sp*_2_ was 0.81 (0.1) and of *sp*_3_ was 0.62 (0.09). The variation in size and aspect ratios *sp*_2_ and *sp*_3_ across the segmented BMCs clearly suggests variability in their gross makeup, although the shape changes could also be due to deformation during image acquisition or reconstruction.

### Classification with simulated data

The key result of this paper is that average overall misclassification error *m*(*Θ*) is small (< 0.04) for a typical polyhedral shape *Θ* in our library $\mathcal{P}$ (Fig. 5b). However, it can be larger (upto 32 %) for some solids. As expected, the misclassification error increases with the degree of truncation.

### Classification with real data

We found an exact match between the topological profiles of observed BMCs and TTPs generated from the library $\mathcal{P}$ for only 7/30 cases. This prompted us to examine other classification methods which are not reliant on an exact match, but least distance from training data. To this end, we considered two commonly used classification procedures, linear discriminant analysis (LDA) [25] and support vector machines (SVM) [26]. The idea is to use the *TTP*s generated from $\mathcal{P}$ as a training set (with labels) to develop decision rules and to evaluate them on the independent test set, as done for the Bayes classifier. Implementing the SVM classifier with on all solids presented a computational problem, as the space and time requirements increase rapidly with training set size [27]. We therefore restricted the SVM computation to all solids with 20 vertices or less, with the justification that the maximum number of observed vertices in BMCs was 10 and for the Bayes classifier, *M*_*Θ* ',*Θ*_ < 0.003 if *Θ* has > 20 vertices, while *Θ*’ has 10 or less. To assess the performance of the SVM and LDA classifier, we compute their regret function, relative to the optimal Bayes classifier, as *r*_*SVM*_(*Θ*) = *m*_*SVM*_(*Θ*) − *m*_*Bayes*_(*Θ*) and analogously for LDA. The mean (SD) regret over the 54 solids in the library $\mathcal{P}$ with 20 vertices or less, was 0.004 (0.01) for SVM and 0.15 (0.12) for LDA at 20 % truncation. This suggests that SVM may be able to deliver very similar performance to the Bayes classifier, with the added advantage of not requiring an exact profile match.

### Robustness of results

Given the large number of processing steps involved in obtaining the PG from raw cryo-ET data, it is possible that the PG contain some errors. We have examined each step of the processing pipeline to examine their impact, as outlined below.

### Application to simulated structures

We have established that with simulated polyhedral structures, we can achieve very accurate classification. To do this, we generated 3-d mesh models of polyhedra from our library of 128 structures (Additional file 2: Figure S8 (a)-(d)). The polyhedron was truncated in at either end in one orientation to mimic the effect of uneven resolution due to reconstruction. Each truncated polyhedron model was sliced in 3 orthogonal directions. Segmentation was performed on each slice using an automated edge detection algorithm in MATLAB. Unlike real data, manual segmentation was not necessary due to the improved contrast resolution. The 3-d object was then ‘reconstructed’ from the segmented boundaries using the volume rendering algorithm we have developed. Subsequently, we manually identified the edges and vertices in the form of a ‘ball and stick diagram’ (Additional file 2: Figure S8 (e)) to construct the incomplete polyhedral graph (PG) of the structure. When we applied our SVM based classification algorithm to the features extracted from this PG, we obtained classification accuracy which was similar to that shown in Additional file 2: Table S2.

### Robustness in image processing

With real data, when there is no independent determination of the structure available, it is not possible to externally validate the final classification result. Instead, we internally validate intermediate steps in the pipeline and examine the sensitivity of our results to perturbations in the data or tuning parameters of the algorithm. The first step we examined was tomographic reconstruction. Here we examined the sensitivity to ‘slice thickness’, a key tuning parameter which controls the resolution the reconstructed volume in the IMOD eTOMO module (see http://bio3d.colorado.edu/imod/doc/etomoTutorial.html for details). We found acceptable contrast resolution for slice thickness values in the range from 200 to 500 slices (in steps of 100). The second step we examined was segmentation and volume rendering: here we examined the sensitivity of volume rendering to using data from only 1 or 2 orthogonal directions as opposed to 3. We observed that while the rendering was unaffected in regions close to the sample plane, there was an appreciable effect in regions affected by the missing wedge. The third step we examined was visual identification of the polyhedral graph. Here we made use of the interdependence between vertices, edges and faces of a polyhedron. Where vertices were identified, we cross-checked that they were connected to at least 3 edges and when both vertices and edges were identified, we performed principal component analysis to ensure that no two adjacent faces were co-planar.

We examined the sensitivity of our methods to object size and image resolution. For this purpose, we applied our method to another dataset based on a different type of BMC, but acquired with the same instrument as the dataset presented here. This new dataset had a pixel size of 12.24 Å × 12.24 Å, while a typical central slice was approximately 800 Å in diameter. By way of comparison, the data set we analysed with the recombinant BMCs had a pixel size of 9.62 Å × 9.62 Å and typical central slice diameter of approximately 1300 Å. Although the contrast resolution of edges was similar in both datasets, we found that it was much harder to identify vertices and edges from the volume renderings of objects in the new dataset due to relatively smaller size. For the same reason, we excluded smaller BMCs from analysis in the dataset presented here. This sensitivity analysis highlights a critical limitation of the proposed methodology, in that it can only be applied to objects that are sufficiently large (> 1100 Å), particularly in the type of imaging setup used for here.

### Robustness to mis-specification in the PG

The fact that there are so few exact matches between the *TTP* from the BMC and the *TTP* of the model polyhedra suggests that there may be errors in the BMC *TTP*s. Given the large number of processing steps required to obtain the PG, there are many potential points at which errors could arise. Ideally, we would like the classification procedure to be robust with respect to such errors. Due to our method for validating marked edges, it is more likely that we might miss a vertex or edge than to identify one that doesn’t exist. To examine robustness, we introduced perturbations in the test set profiles by randomly deleting a vertex and linked edges from the truncated polyhedron and recomputing the corresponding *TTP*. Note that most *TTP* features are affected by this deletion. The training data still consisted of the original *TTP*s. As very few exact *TTP* matches were found in the test set, so *m*_*Bayes*_(*Θ*) ≈ 1. At 20 % truncation, the deterioration in *m*_*SVM*_(*Θ*) was more modest (mean of 0.25 and max of 0.54) (Fig. 5c), while the corresponding *m*_*LDA*_(*Θ*) had a mean of 0.36 and max of 0.89. Detailed analysis of the confusion matrix *M*_*Θ* ',*Θ*_, shows that even with a mis-specified PG, the chance of misclassifying a Platonic solid as a Johnson solid is very small (< 0.054, Additional file 2: Table S1).

### The importance of global, local and incomplete topology

We computed classification rules based solely on feature subsets defined in Table 1. For correctly specified truncated topological profiles, just the local and complete features appear to be similarly accurate as all features (Fig. 5c). However, when the PG is mis-specified, i.e. with random vertex dropped, the importance of incomplete and global features becomes more apparent, as the average regret for complete features is 0.06 and for local features is 0.04 (Fig. 5d). These figures also illustrate the inadequacy of the global features alone in accurately classifying from truncated polyhedra.

### Application to micro-compartment data

**Fig. 6:**
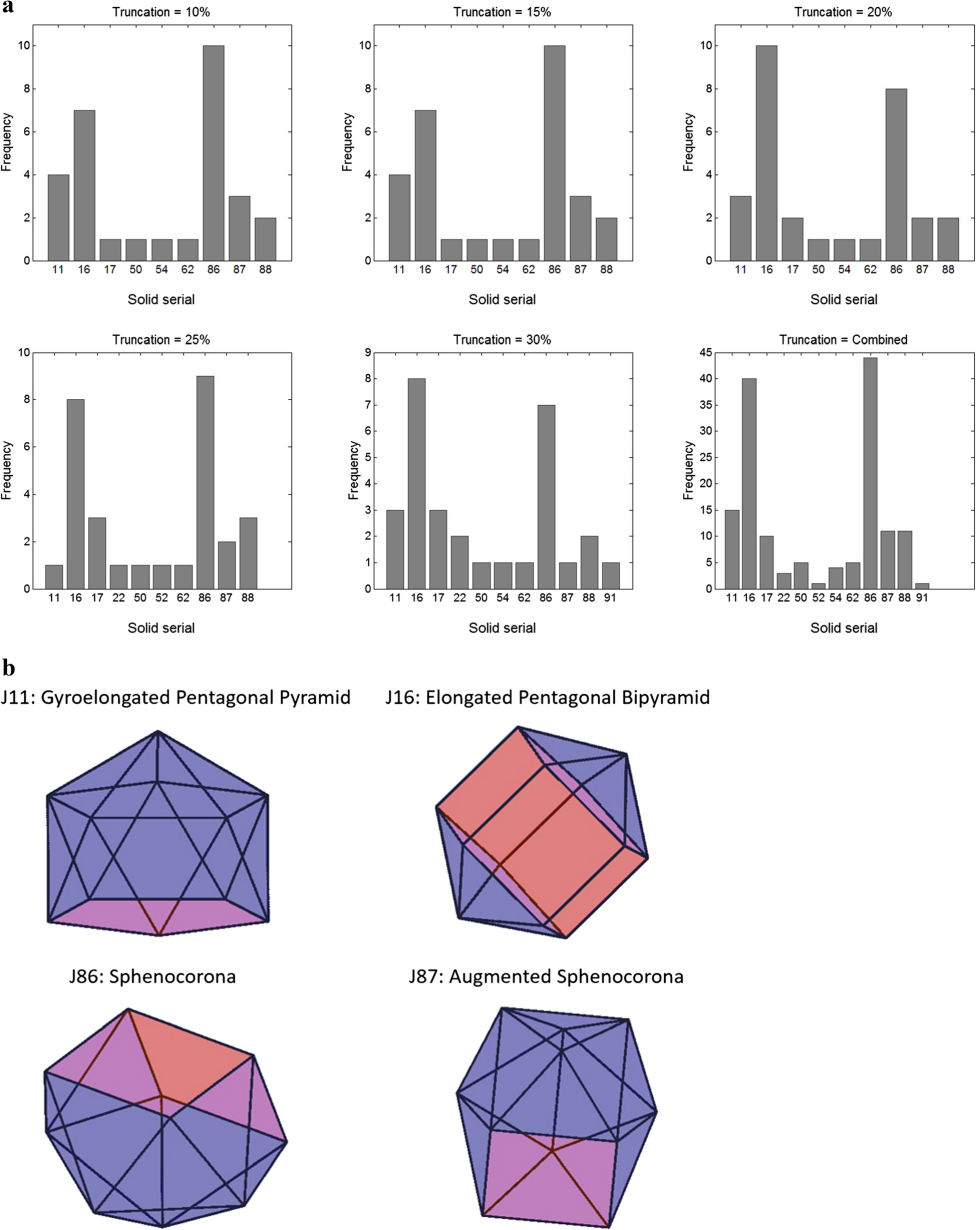
**a** Distribution of predicted polyhedra for 30 E. coli micro-compartments for different levels of assumed truncation. Names of solids corresponding to the serial numbers are given in Additional file 3: Table S3. All belong to the Johnson solids family. **b** Volume renderings of most commonly predicted solids

The solids predicted by the SVM classifier for 30 BMCs were all Johnson solids, with the most common being the sphenocorona (J86) and the elongated pentagonal bipyramid (J16) (Fig. 6b, Additional file 1: Table S4 and Additional file 3: Table S3). The consistency in predictions across assumed levels of truncation *t* and *s, =* 10 %, 15 %, 20 %, 25 % or 30 %, *t ≠ s*, was measured using the estimated match probability ${p}_{t,s}^M={30}^{-1}{\displaystyle {\sum}_{j=1}^{30}I\left({\widehat{\varTheta}}_{j,s}\ne {\widehat{\varTheta}}_{j,t}\right)}$, where ${\widehat{\varTheta}}_{i,s}\ \mathrm{and}\ {\widehat{\varTheta}}_{i,t}$ are the respective predicted polyhedra. The mean (SD) for *p*_*t*,*s*_^*M*^ was 0.81 (0.12), suggesting good agreement, although it expectedly decreased as a function of |*t – s*|. The positive predictive value was estimated as PPV = $P\left(\left.\varTheta \right|\widehat{\varTheta}\right)=1-{M}_{\varTheta, \widehat{\varTheta}}/{\displaystyle {\sum}_{\varTheta \in \mathcal{P}}{M}_{\varTheta, \widehat{\varTheta}}}$. At 20 % truncation, the estimated average PPV based on mis-specified (random vertex deletion) test PGs was 0.7 (Additional file 2: Table S2), suggesting moderately high confidence in the predictions.

## Methodological discussion

We have demonstrated that it is possible to accurately classify polyhedral shapes from truncated versions using features of their polyhedral graph (PG) within a large library of polyhedra. There are a few shapes which are harder to classify, because their PG is either identical or very closely resembles the PG of another polyhedral shape in the class. For typical solids, the degree of accuracy is minimally affected by the degree of truncation, within a range of 10 % to 30 % truncation. Even when the PG is mis-specified by randomly dropping vertices, most shapes in the library can be classified with reasonable accuracy. In conjunction, these results establish the feasibility of predicting polyhedral shapes from individual incomplete 3-D reconstructions obtained using cryo-ET. Our results indicate that even when two of these BMC share a common polyhedral structure, they can vary widely in size, aspect ratios (Additional file 2: Figure S9). Further there is significant variation in edge lengths within a face. Together, these imply that there is no straightforward way to appropriately align any pair of these BMCs. Consequently physical averaging of sub-tomograms, as a strategy to improve resolution, is not feasible here. This result emphasizes the utility of a single object analysis approach for studying heterogeneity of shape and asymmetry, both of which might be masked by sub-tomogram averaging.

Our results with simulated polyhedral structures indicate that the pipeline accurately identifies the true polyhedral structure when high resolution data is available. We have introduced various cross-checks to ensure that the PG is accurately identified. To guard against possible errors that might creep in despite these chesk, we have examined the impact of deleting a random vertex on the sensitivity of the classification process: it does result in a substantial increase misclassification error. Even in this setting, support vector machine based classification appears to yield reasonable misclassification error rates for most polyhedral shapes. We therefore recommend using the SVM based classifier over either the exact Bayes classifier or linear discriminant analysis for this application. For large scale processing and observer independent results, it is desirable that the entire pipeline is automated. With real data, we found that many standard automated methods for segmentation of object boundaries or recognition of edges produced unacceptable levels of spurious results. Further work is required in developing automated methods for detecting edges in 2 and 3-d, which are suited to the complex texture of cryo EM reconstructions.

We also developed an alternative approach to simpler method for identifying shapes from incomplete polyhedra, based on extending partially visible edges and predicting missing vertices based on their points of intersection (Chapter 5 of [19]). The results from that approach also suggest that the shapes belong to the Johnson solids family. However, we prefer the classification based approach, because it allows us to quantify the uncertainty of prediction. An important limitation of the proposed method is that the single object analysis could only be applied to objects that are sufficiently large in maximal cross-sectional diameter (> 1100 Å). More broadly, the method is potentially applicable to any macromolecular structure where a polyhedral structure is suspected, such as virus caspids.

## Conclusion

Results for recombinant micro-compartments (BMC) in *E.coli.* indicate that they are all similar to Johnson solids in shape: these are non-symmetric polyhedra (Additional file 4: Figure S10). Secondly, the predicted shapes were not all identical, though two shapes predominated. Our sensitivity analysis indicates that even though there may be uncertainty about the exact polyhedral structure of a particular BMC (some Johnson solids are quite similar to each other), we can confidently rule out the possibility that they have a symmetric shape, i.e. a Platonic solid. This result contrasts with previously published results for BMCs from other types of bacteria, which suggest a unique symmetric icosahedral shape [6, 28]. For molecular structures generated from inhomogeneous elastic shells, i.e. those composed of at least two types of molecules, such as BMCs, Vernizzi et al. [29] have argued, using simulations based on minimum energy principles, that such shells should spontaneously buckle into non-symmetric polyhedral shapes within the Johnson family. Further they have argued that the exact shape would vary somewhat randomly. Our results provide empirical validation of these simulation based results. The heterogeneity of shape suggests two possible hypotheses; a) there might be a diversity of functionality between the differently shaped BMCs b) non-symmetric shapes may have been favored by evolution because they provide a more optimal surface for catalytic operations than a symmetric polyhedral or spherical shell. Further work will be required to test these hypotheses.

## Additional files

Additional file 1: Table S4. (in separate Excel spreadsheet due to size): List of estimated polyhedral graphs for 30 micro-compartments of *E. coli*, as represented by their topological profiles. (XLSX 33 kb) Additional file 2: Figure S1. Distribution of number of edges per face for Platonic solids. **Figure S2.** Distribution of vertex degree (number of edges meeting at a vertex) for Platonic solids. **Figure S3.** Distribution of vertex degree for Archimedean solids. **Figure S4.** Distribution of number of edges per face for Archimedean solids. **Figure S5.** Distribution of vertex degree for 6 Johnson solids. **Figure S6.** Distribution of number of edges per face for 6 Johnson solids. **Figure S7.** The distribution of vertex degree in MCs. **Figure S8.** Simulated standard Polyhedron and their view after truncation - (a) A simulated icosahedron, (b) the icosahedron with missing top, (c) A simulated sphenocorona, (d) the sphenocorona with missing top and (e) the ball-stick diagram on (d). Figure S9: Distribution of aspect ratios of reconstructed BMCs by identified shape. **Table S1.** Test set misclassification error for SVM classifier summarised by class of solid. This analysis is based on the set of 54 solids with 20 vertices or less. **Table S2.** Predicted polyhedral shapes for 30 E. coli microcompartments using the SVM classifier. The names of the solids corresponding the serial numbers are given in **Table S3** and all belong to the Johnson solids family. The positive predictive value (PPV) is the chance that the correct solid was identified, based on estimated misclassification errors obtained using a mis-specified polyhedral graph test set. **Table S5.** Categorization of features in the topological profile (TP) of a polyhedral graph (PG). (PDF 702 kb) Additional file 3: Table S3. (in separate Excel spreadsheet due to size): List of 123 polyhedra in the library $\mathcal{P}$, together with class and topological profiles (TP). Polyhedral pairs with identical TP are noted. (XLSX 99 kb) Additional file 4: Figure S10. (In separate file titled: 2D view of complete tomograms.pdf) Tomograms showing objects selected for reconstruction. (PDF 4567 kb) Additional file 5: Figure S11. (In separate file titled: Individual BMC Shapes.pdf) 3-d volume renderings of individual reconstructed BMCs, followed by 3-d volume renderings of identified polyhedral shapes. (PDF 470 kb)
