# Supplementary material for: Classification of polyhedral shapes from individual anisotropically resolved cryo-electron tomography reconstructions
Source: BMC Bioinformatics. 2016 Jun 13;17:234. doi: 10.1186/s12859-016-1107-5 (PMC4904361; doi:10.1186/s12859-016-1107-5)
Supplement: Additional file 2: Figure S1. — Distribution of number of edges per face for Platonic solids. Figure S2. Distribution of vertex degree (number of edges meeting at a vertex) for Platonic solids. Figure S3. Distribution of vertex degree for Archimedean solids. Figure S4. Distribution of number of edges per face for Archimedean solids. Figure S5. Distribution of vertex degree for 6 Johnson solids. Figure S6. Distribution of number of edges per face for 6 Johnson solids. Figure S7. The distribution of vertex degree in MCs. Figure S8. Simulated standard Polyhedron and their view after truncation - (a) A simulated icosahedron, (b) the icosahedron with missing top, (c) A simulated sphenocorona, (d) the sphenocorona with missing top and (e) the ball-stick diagram on (d). Figure S9: Distribution of aspect ratios of reconstructed BMCs by identified shape. Table S1. Test set misclassification error for SVM classifier summarised by class of solid. This analysis is based on the set of 54 solids with 20 vertices or less. Table S2. Predicted polyhedral shapes for 30 E. coli microcompartments using the SVM classifier. The names of the solids corresponding the serial numbers are given in Table S3 and all belong to the Johnson solids family. The positive predictive value (PPV) is the chance that the correct solid was identified, based on estimated misclassification errors obtained using a mis-specified polyhedral graph test set. Table S5. Categorization of features in the topological profile (TP) of a polyhedral graph (PG). (PDF 702 kb) [file 12859_2016_1107_MOESM2_ESM.pdf]

Supplementary information for : Classification of polyhedral shapes from individual incomplete cryo-EM reconstructions

List of supplementary figures:

Figure S1: Distribution of number of edges per face for Platonic solids

Figure S2: Distribution of vertex degree (number of edges meeting at a vertex) for Platonic solids

Figure S3: Distribution of vertex degree for Archimedean solids

Figure S4: Distribution of number of edges per face for Archimedean solids

Figure S5: Distribution of vertex degree for 6 Johnson solids

Figure S6: Distribution of number of edges per face for 6 Johnson solids

Figure S7: The distribution of vertex degree in MCs

Figure S8: Simulated standard Polyhedron and their view after truncation - (a) A simulated icosahedron, (b) the icosahedron with missing top, (c) A simulated sphenocorona, (d) the sphenocorona with missing top and (e) the ball-stick diagram on (d)

Figure S9: Distribution of aspect ratios of reconstructed BMCs by identified shape.

Figure S10: (In separate file titled: 2D view of complete tomograms.pdf) Tomograms showing objects selected for reconstruction.

Figure S11: (In separate file titled: Individual BMC Shapes.pdf) 3-d volume renderings of individual reconstructed BMCs, followed by 3-d volume renderings of identified polyhedral shapes.

List of supplementary tables:

Table S1: Test set misclassification error for SVM classifier summarised by class of solid. This analysis is based on the set of 54 solids with 20 vertices or less.

Table S2: Predicted polyhedral shapes for 30 E. coli microcompartments using the SVM classifier. The names of the solids corresponding the serial numbers are given in Table S3 and all belong to the Johnson solids family. The positive predictive value (PPV) is the chance that the correct solid was identified, based on estimated misclassification errors obtained using a mis-specified polyhedral graph test set.

Table S3 (in separate Excel spreadsheet due to size): List of 123 polyhedra in the library $\mathcal{P}$, together with class and topological profiles (TP). Polyhedral pairs with identical TP are noted.

Table S4 (in separate Excel spreadsheet due to size): List of estimated polyhedral graphs for 30 micro-compartments of *E. coli*, as represented by their topological profiles.

Table S5: Categorization of features in the topological profile (TP) of a polyhedral graph (PG)

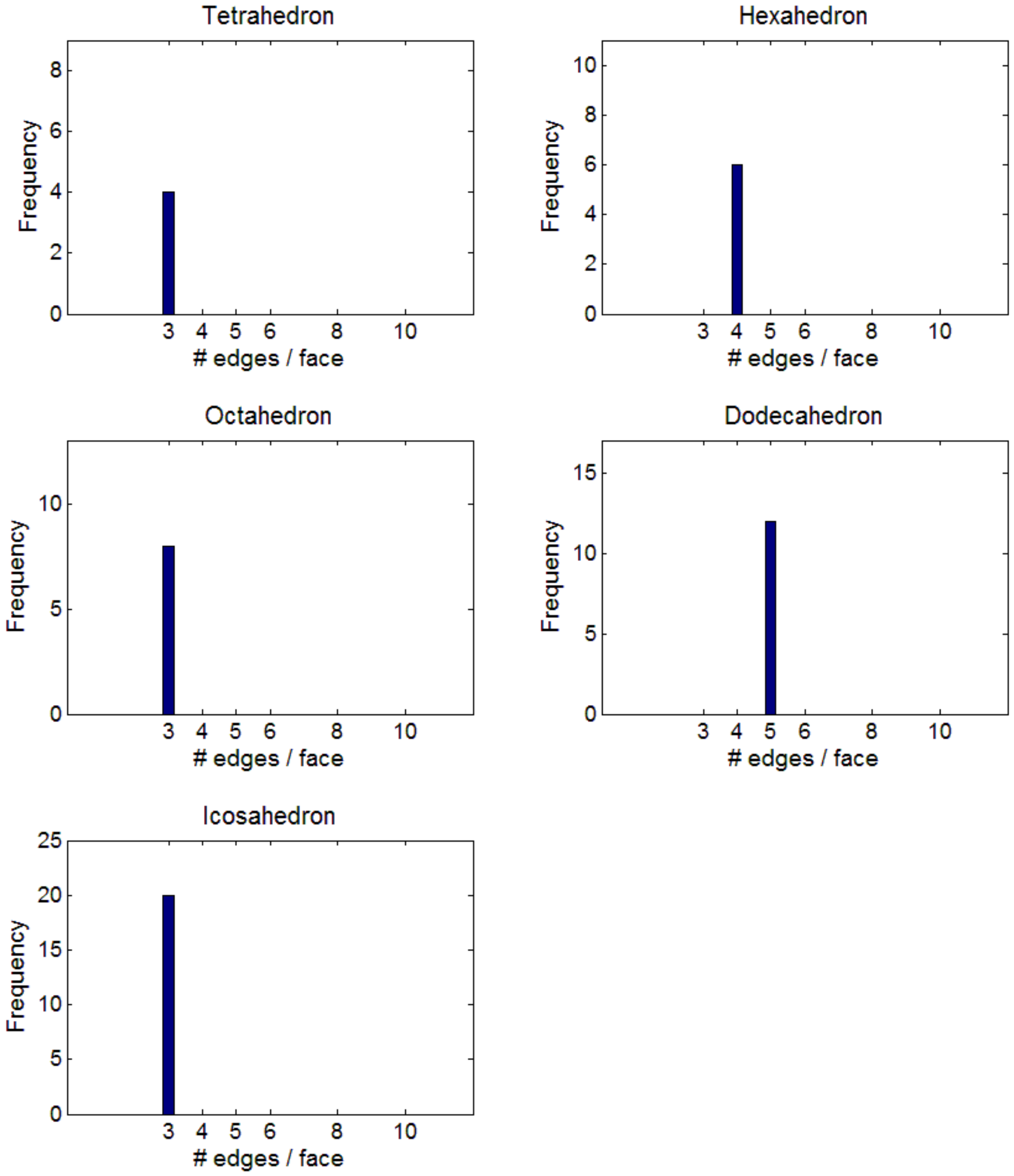

Figure S1: Distribution of number of edges per face for Platonic solids

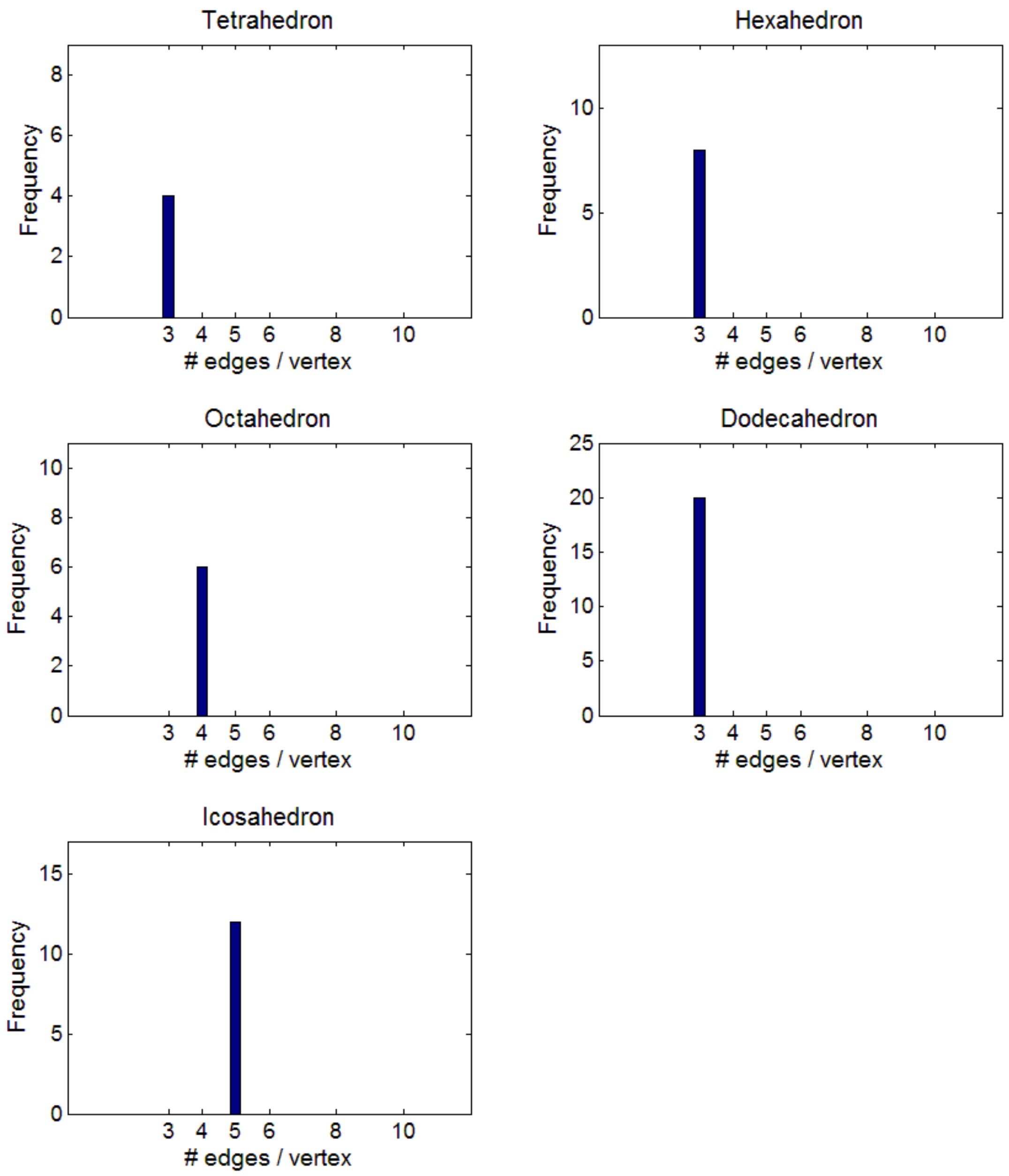

Figure S2: Distribution of vertex degree (number of edges meeting at a vertex) for Platonic solids

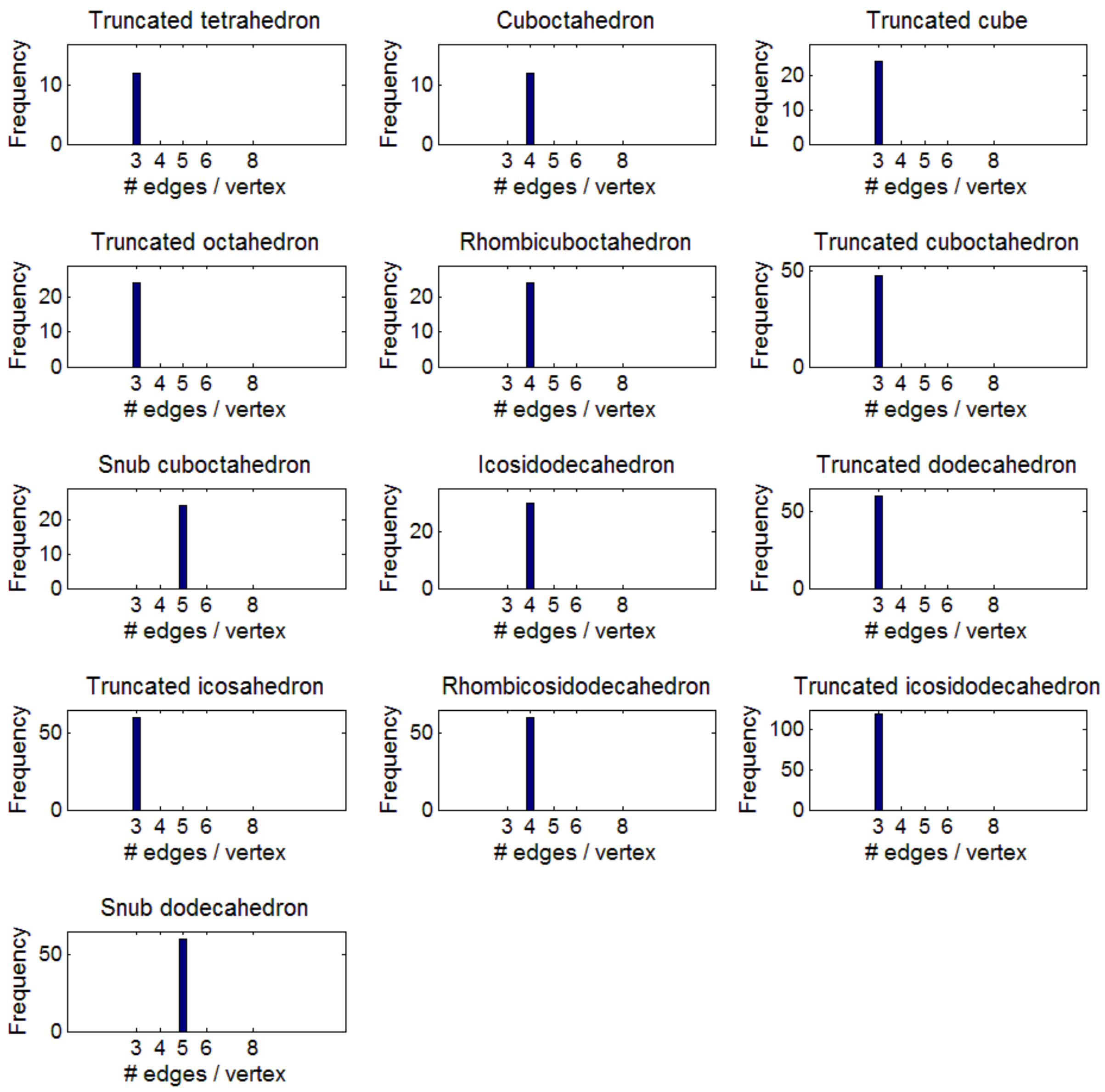

Figure S3: Distribution of vertex degree for Archimedean solids

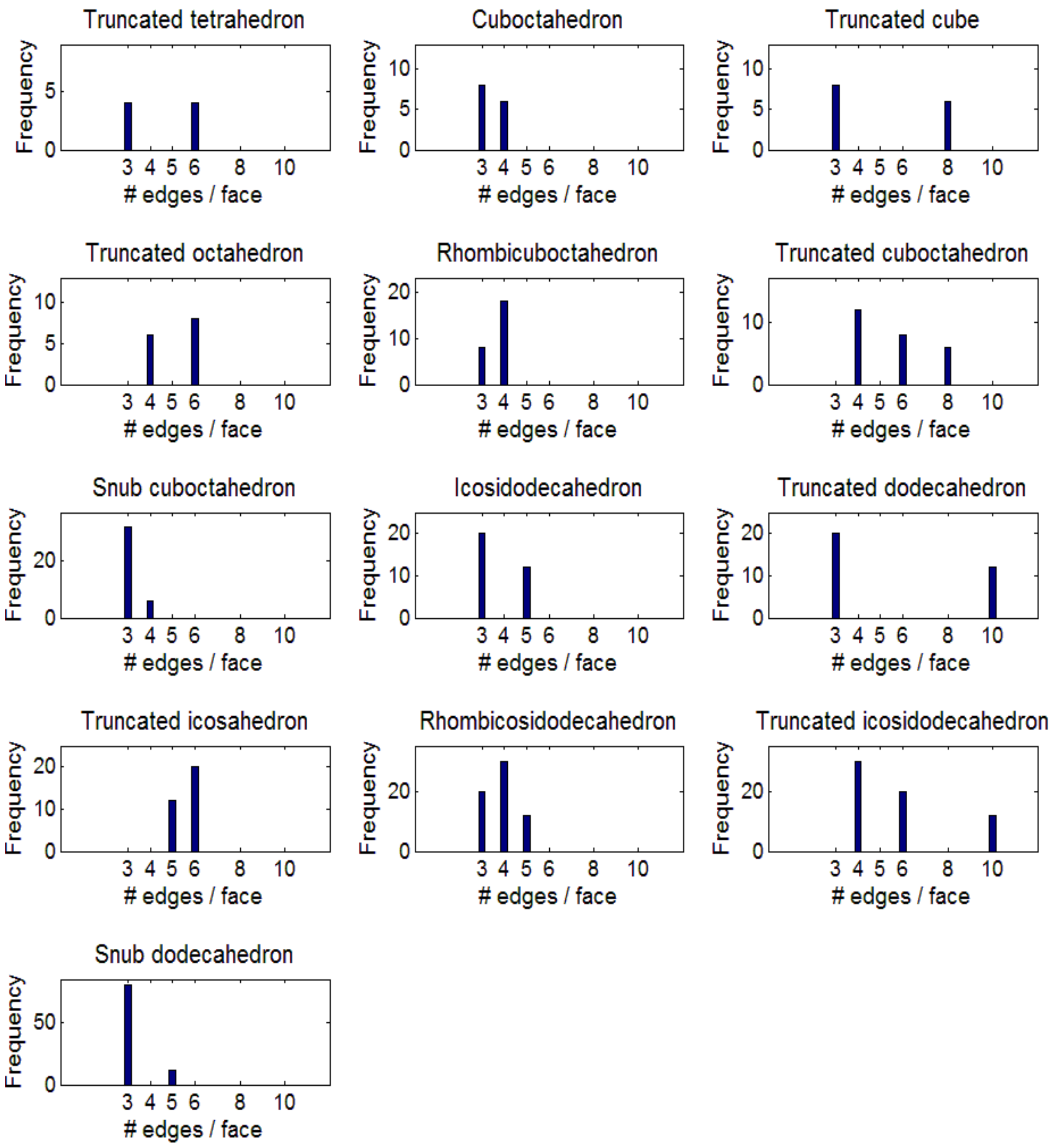

Figure S4: **Distribution of number of edges per face for Archimedean solids**

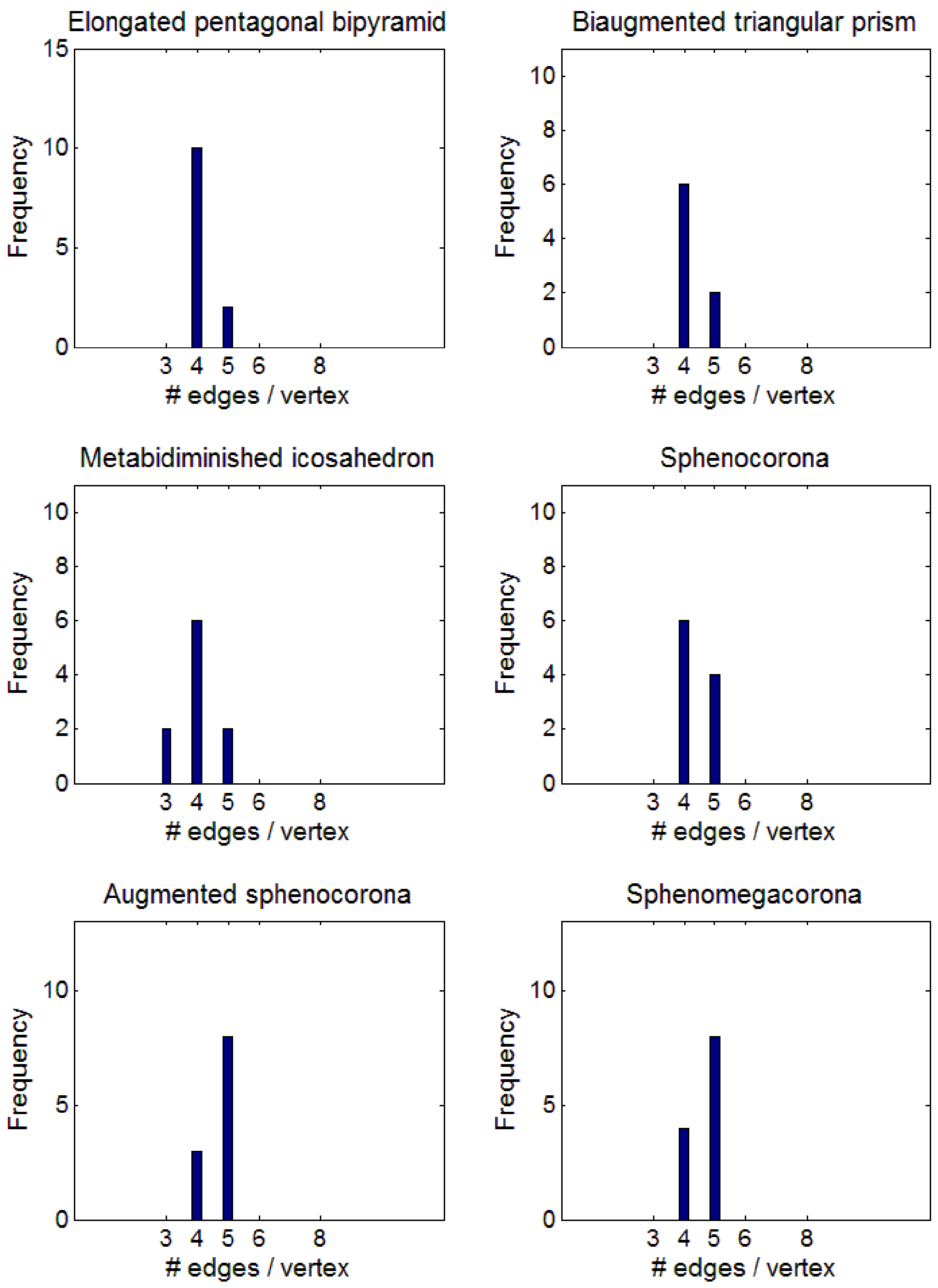

Figure S5: Distribution of vertex degree for 6 Johnson solids

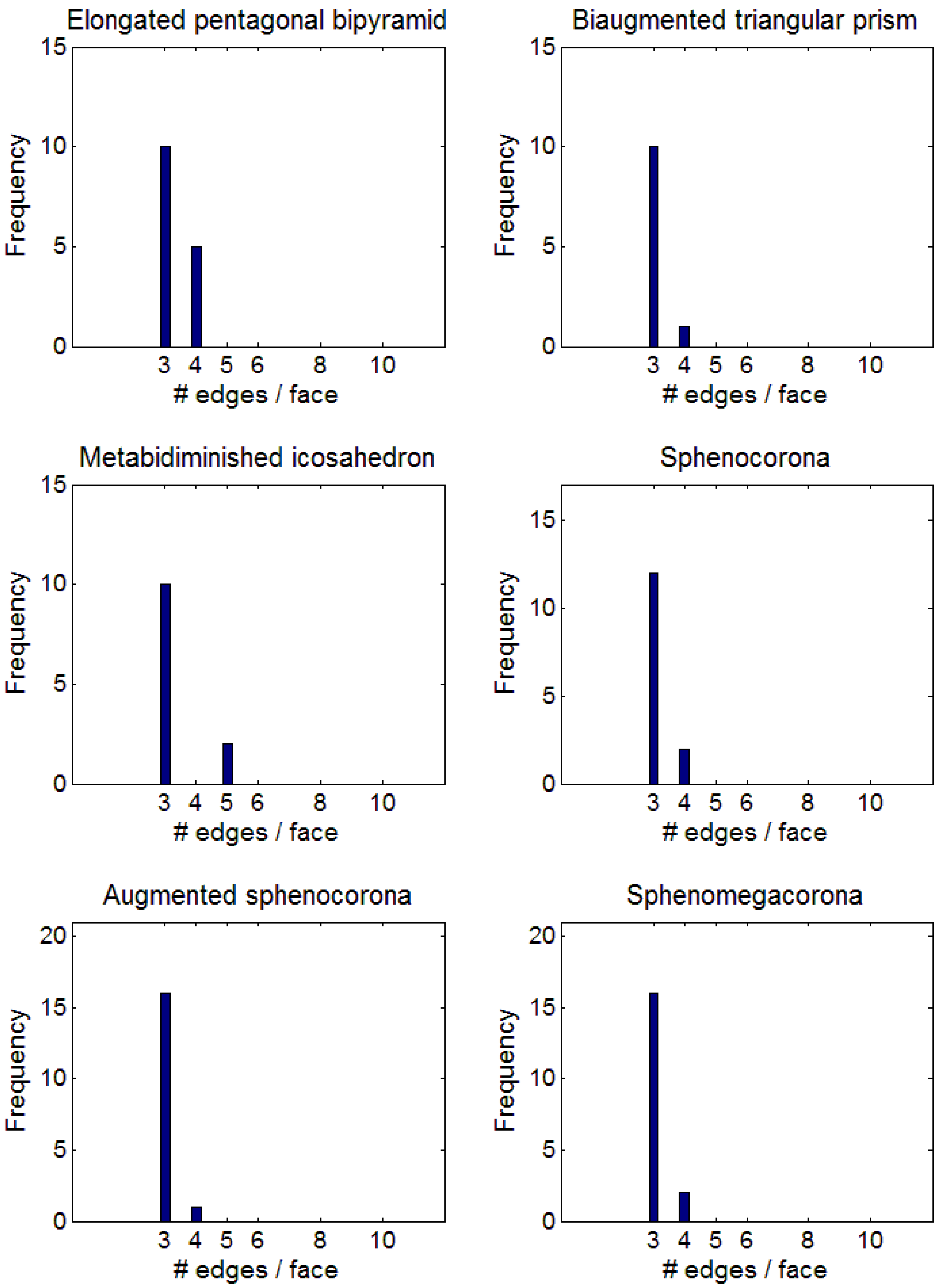

Figure S6: Distribution of number of edges per face for Johnson solids

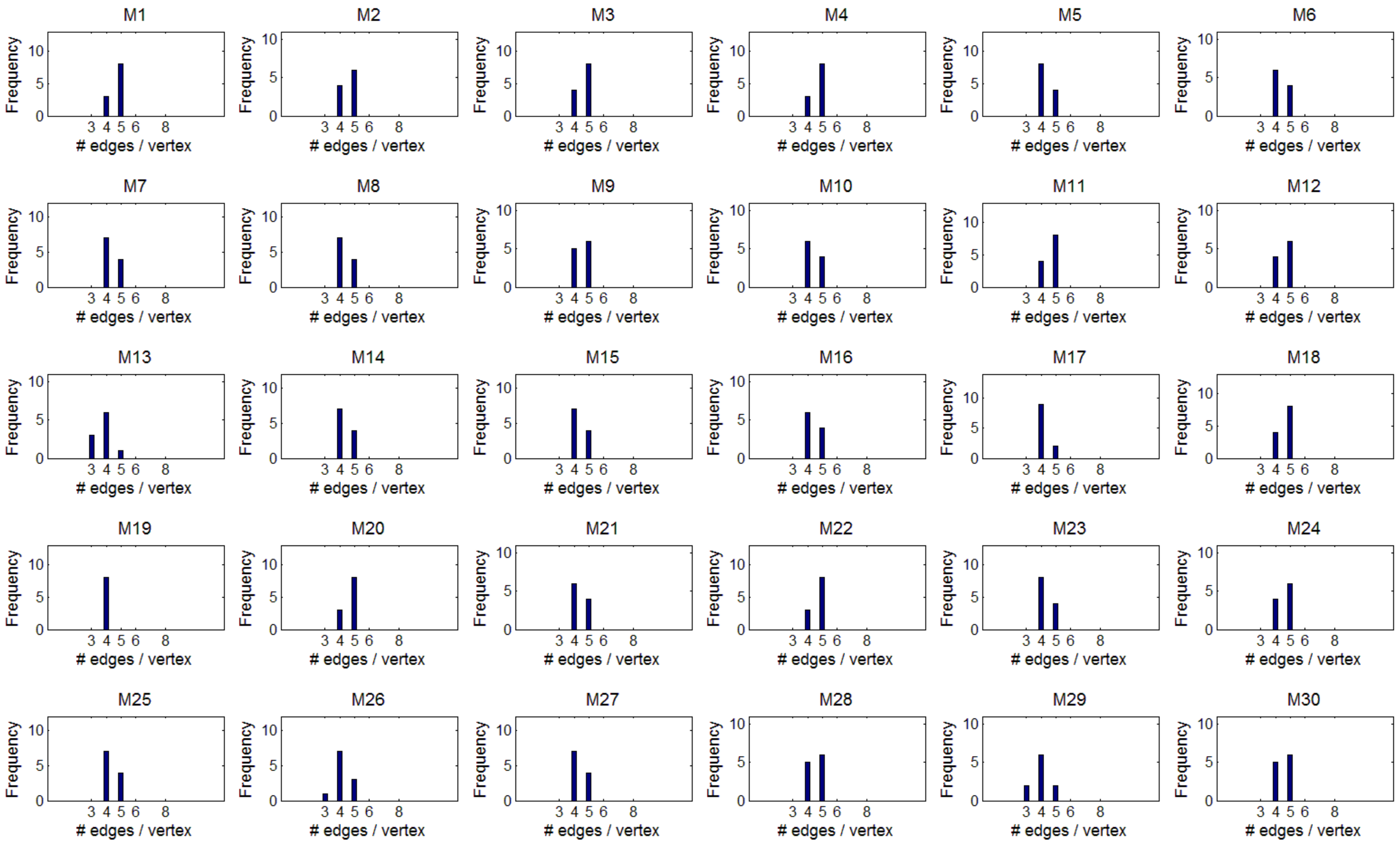

Figure S7: The distribution of vertex degree in MCs

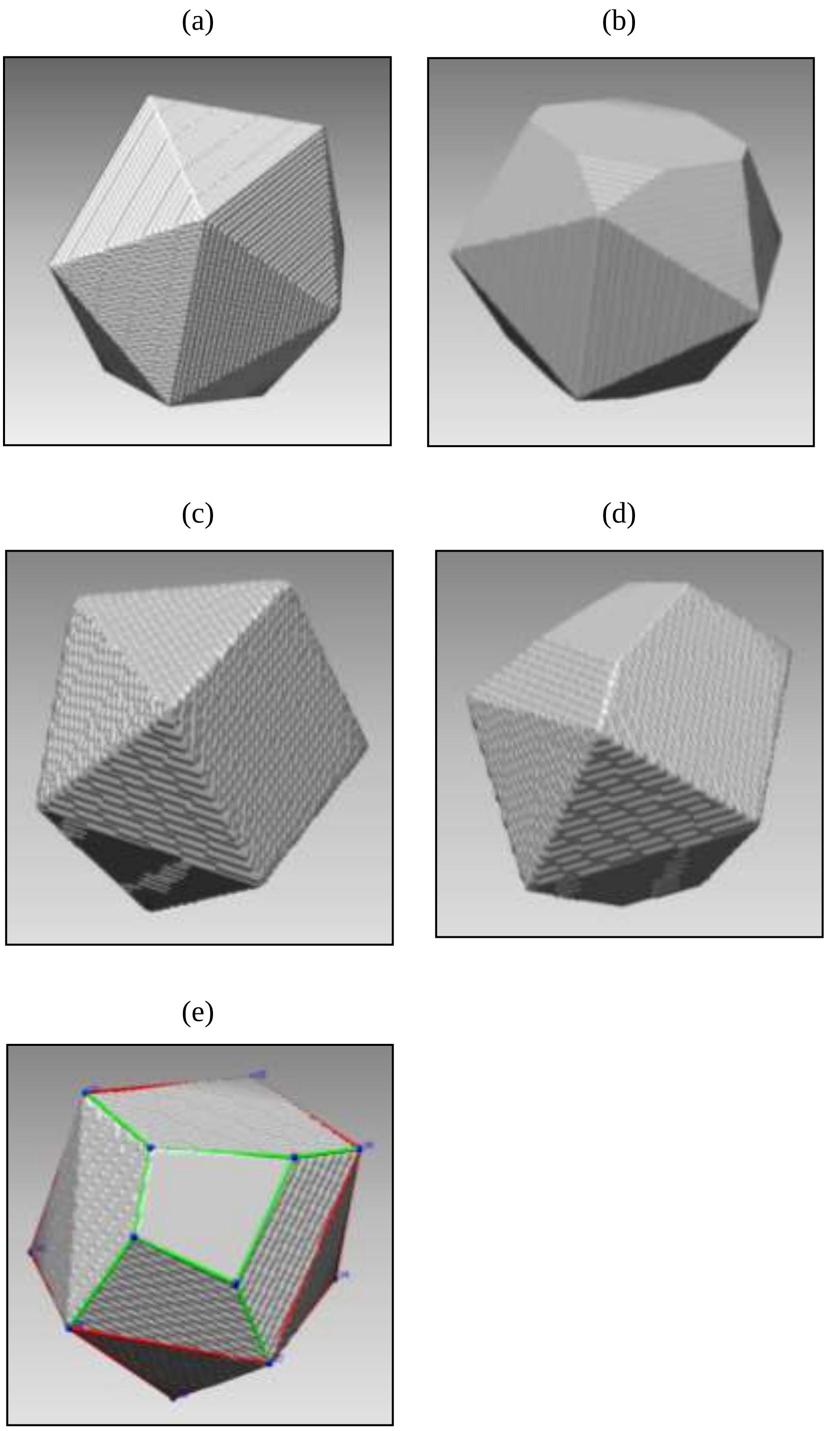

Figure S8: **Simulated standard Polyhedron and their view after truncation - (a) A simulated icosahedron, (b) the icosahedron with missing top, (c) A simulated sphenocorona, (d) the sphenocorona with missing top and (e) the ball-stick diagram on (d)**

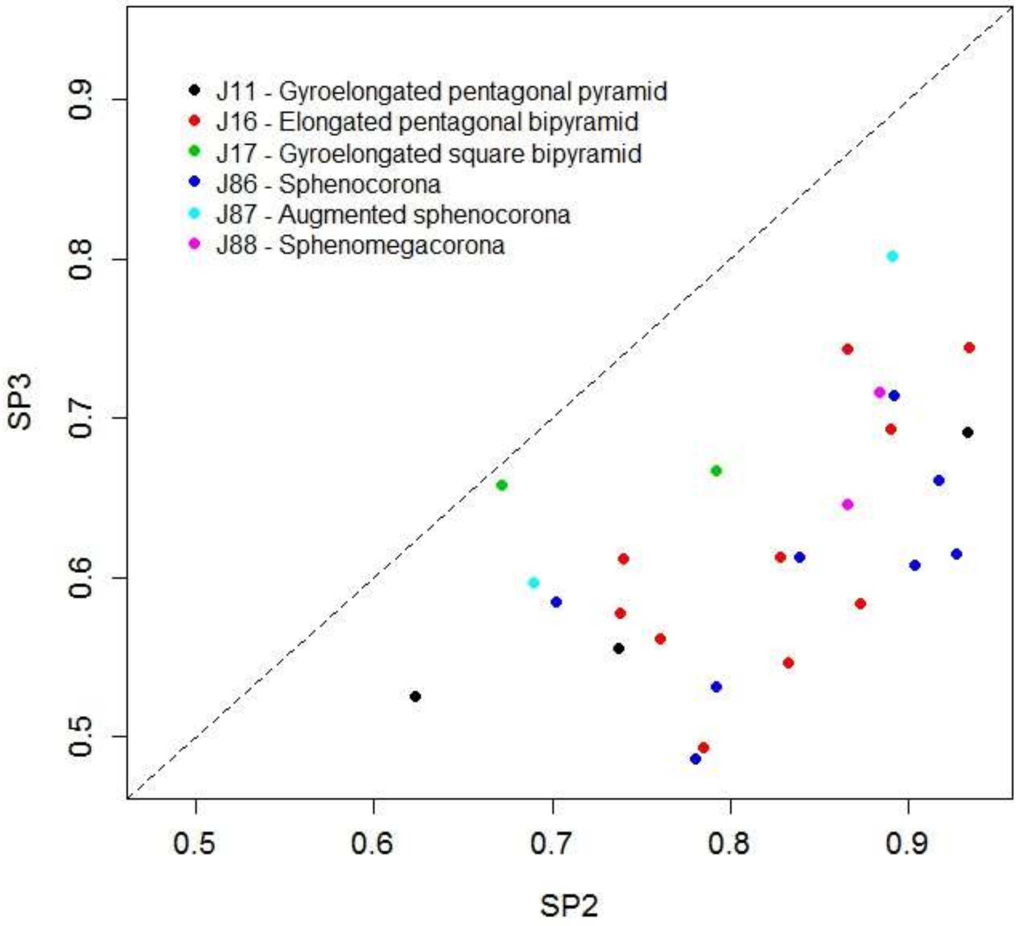

Figure S9: Distribution of aspect ratios by identified structure of BMCs. SP2 = λ_2/λ_1, SP3 = λ_3/λ_1, where $\lambda_1 \geq \lambda_2 \geq \lambda_3$ are the principal axes of an ellipsoid fitted to each reconstructed BMC.

Table S1: Test set misclassification error for SVM classifier summarised by class of solid. This analysis is based on the set of 54 solids with 20 vertices or less. The highlighted section refers to the chance of misclassification of a symmetric Platonic solid as a Johnson solid.

		Actual shape class			
**Predicted shape class**	**Misclassification error**	**Johnson Solids**	**Platonic Solids**	**Archimedean Solids**	**Catalan Solids**
**Johnson Solids**	Minimum	0.0120	0.0000	0.0200	0.0000
	Maximum	0.5240	0.0540	0.4620	0.0020
	Median	0.2680	0.0320	0.2410	0.0000
	Mean	0.2763	0.0287	0.2410	0.0005

	SD	0.1462	0.0272	0.3125	0.0010
	5th Percentile	0.0520	0.0000	0.0200	0.0000
	95th Percentile	0.4990	0.0540	0.4620	0.0020
**Platonic Solids**	Minimum	0.0000	0.0000	0.0000	0.0000
	Maximum	0.0320	0.3200	0.2900	0.0000
	Median	0.0000	0.0320	0.1450	0.0000
	Mean	0.0014	0.1173	0.1450	0.0000
	SD	0.0063	0.1762	0.2051	0.0000
	5th Percentile	0.0000	0.0000	0.0000	0.0000
	95th Percentile	0.0085	0.3200	0.2900	0.0000
**Archimedean Solids**	Minimum	0.0000	0.0000	0.0040	0.0000
	Maximum	0.3960	0.0020	0.5400	0.0000
	Median	0.0000	0.0000	0.2720	0.0000
	Mean	0.0120	0.0007	0.2720	0.0000
	SD	0.0603	0.0012	0.3790	0.0000
	5th Percentile	0.0000	0.0000	0.0040	0.0000
	95th Percentile	0.0520	0.0020	0.5400	0.0000
**Catalan Solids**	Minimum	0.0000	0.0000	0.0000	0.0000
	Maximum	0.0380	0.0000	0.0000	0.0340
	Median	0.0000	0.0000	0.0000	0.0160
	Mean	0.0015	0.0000	0.0000	0.0165
	SD	0.0059	0.0000	0.0000	0.0154
	5th Percentile	0.0000	0.0000	0.0000	0.0000
	95th Percentile	0.0080	0.0000	0.0000	0.0340

Table S2: Predicted polyhedral shapes for 30 E. coli microcompartments using the SVM classifier. The names of the solids corresponding the serial numbers are given in Table S3. The positive predictive value (PPV) is the chance that the correct solid was identified, based on estimated misclassification errors obtained using a mis-specified polyhedral graph test set.

Microcompartment No	Predicted Shape (Serial No.)	Positive Predictive Value

		(PPV)
1	17	0.70
2	11	0.69
3	16	0.75
4	11	0.69
5	16	0.75
6	86	0.69
7	86	0.69
8	86	0.69
9	16	0.75
10	86	0.69
11	88	0.59
12	86	0.69
13	54	0.62
14	16	0.75
15	86	0.69
16	86	0.69
17	16	0.75
18	88	0.59
19	50	0.76
20	87	0.60
21	86	0.69
22	87	0.60
23	16	0.75
24	17	0.70
25	16	0.75
26	16	0.75
27	16	0.75
28	16	0.75
29	62	0.81
30	11	0.69
**Mean PPV**		0.70

Table S5: Categorization of features in the topological profile (TP) of a polyhedral graph (PG)

		**Feature type**			
**Topological profile component**	**Dimension**	**Complete**	**Incomplete**	**Global**	**Local**
V,E,F	3	x		x	
Face type distribution	6	x			x

Vertex degree distribution	6	x			x
At least face type distribution	8		x		x
At least vertex type distribution	8		x		x
Edge adjacency matrix	10x10 = 100	x			x
Face adjacency matrix	10x10 = 100	x			x
Total	231				
