# Supplementary material for: Classification of polyhedral shapes from individual anisotropically resolved cryo-electron tomography reconstructions
Source: BMC Bioinformatics. 2016 Jun 13;17:234. doi: 10.1186/s12859-016-1107-5 (PMC4904361; doi:10.1186/s12859-016-1107-5)

2D view of complete tomograms

Tomogram 05

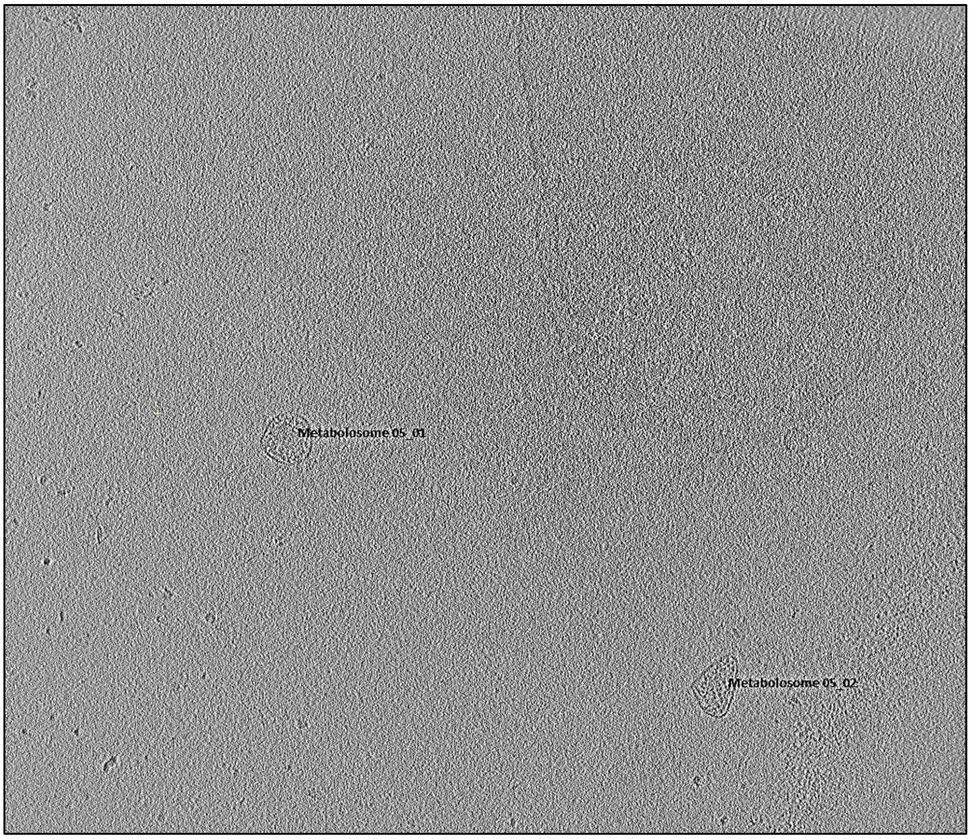

Tomogram 13 (View 1)

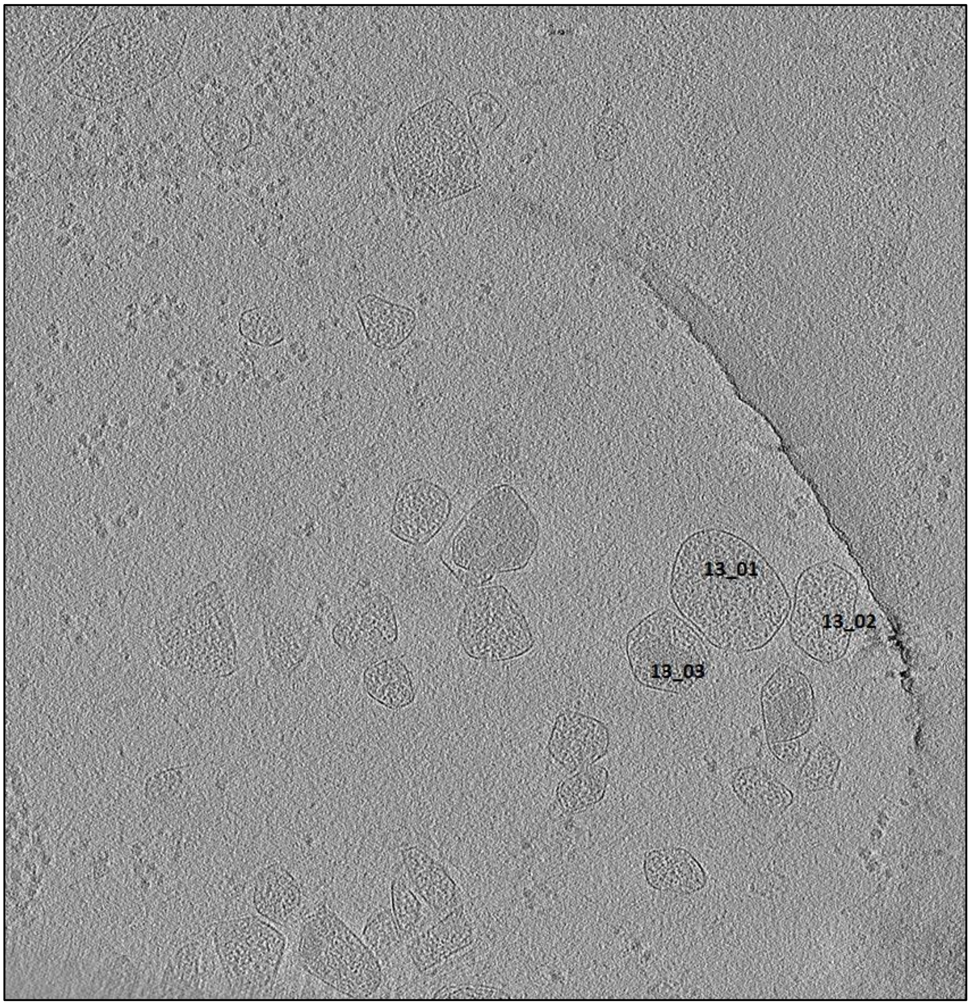

Tomogram 13 (View 2)

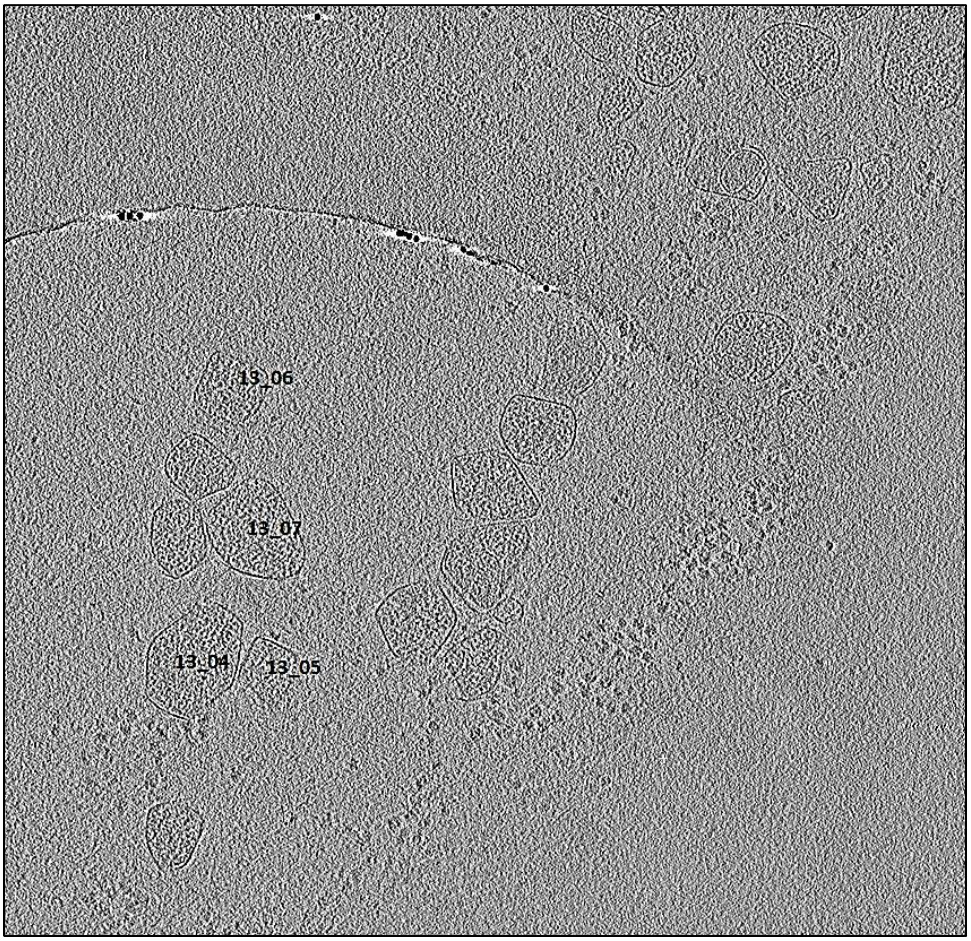

Tomogram 13 (View 3)

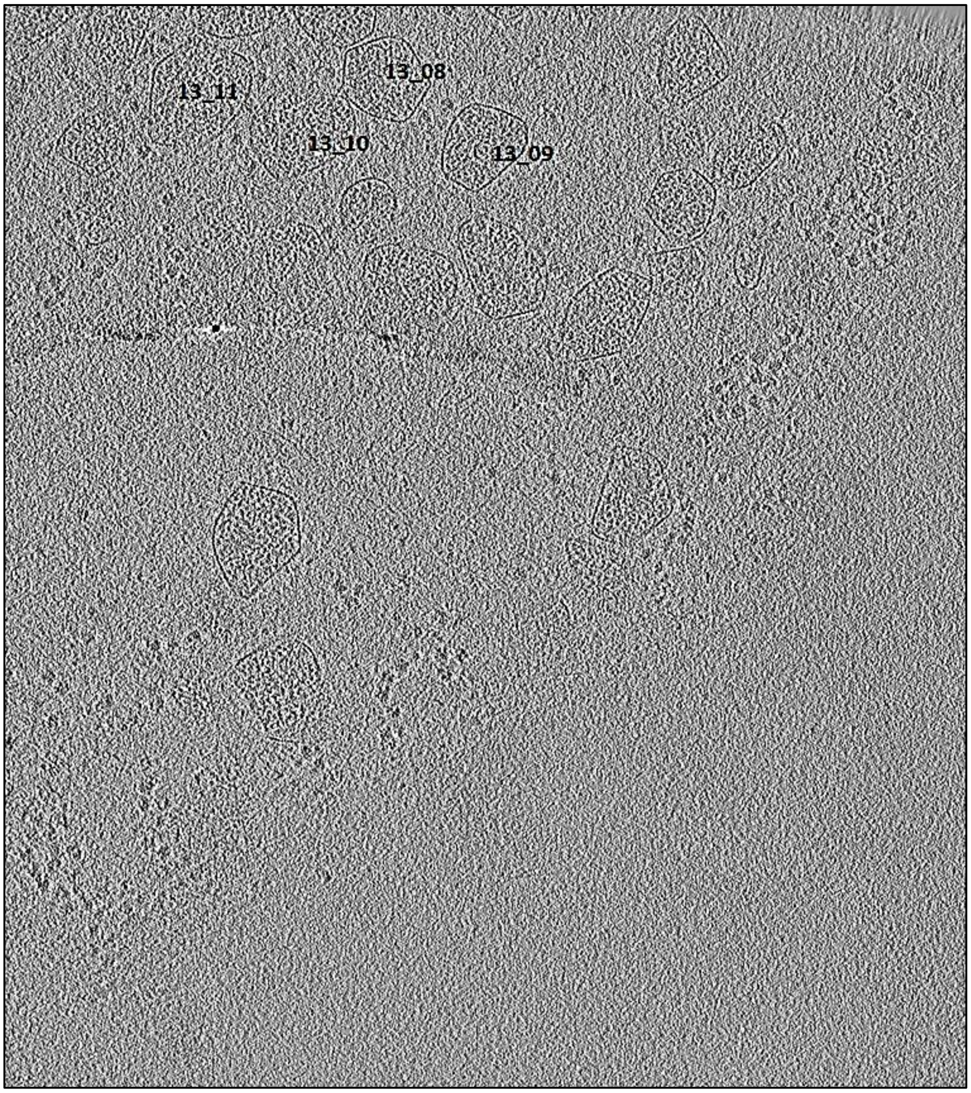

Tomogram 13 (View 4)

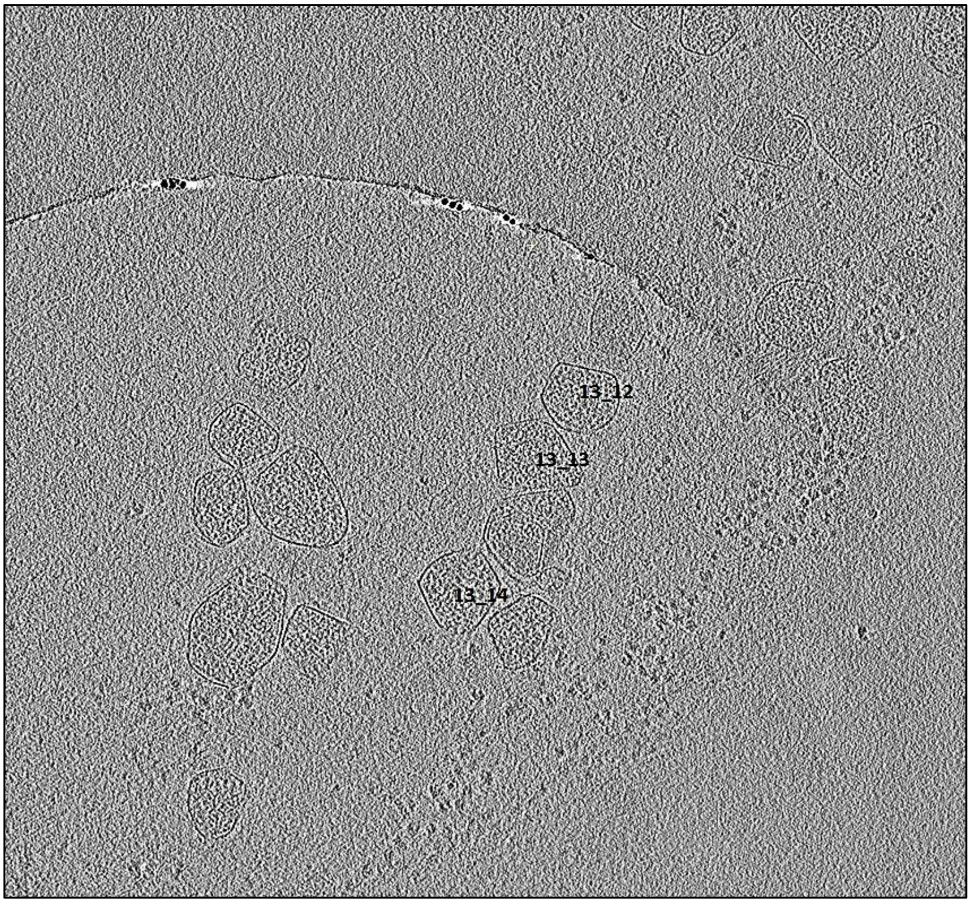

Tomogram 20 (View 1)

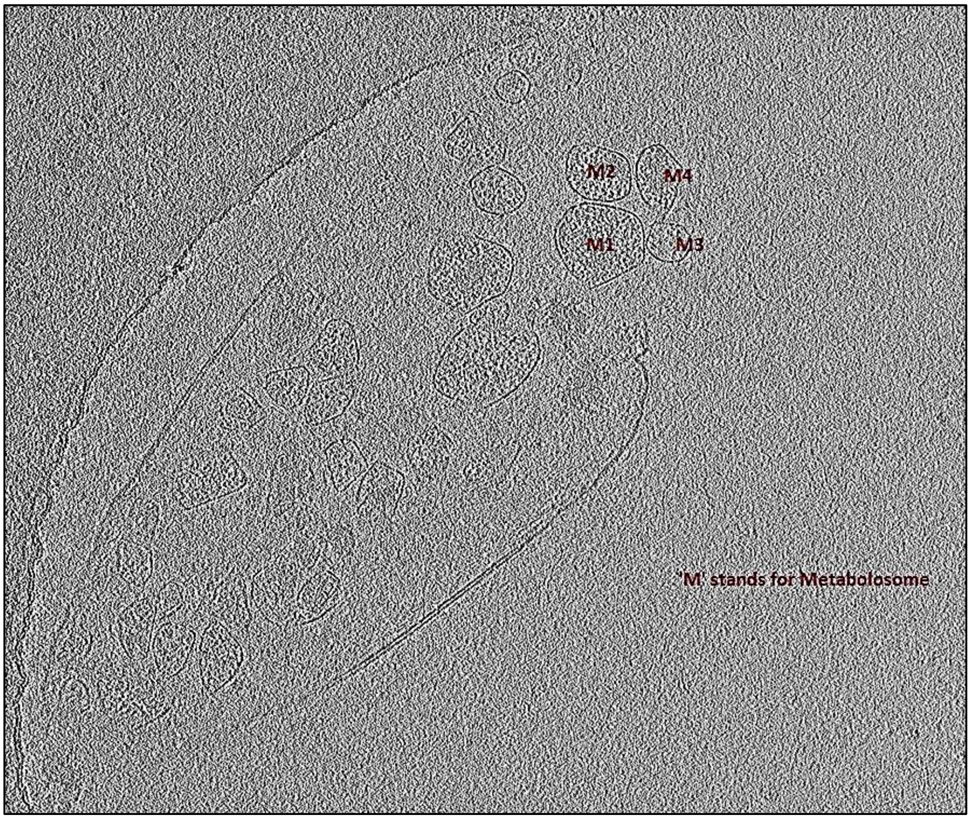

Tomogram 20 (View 2)

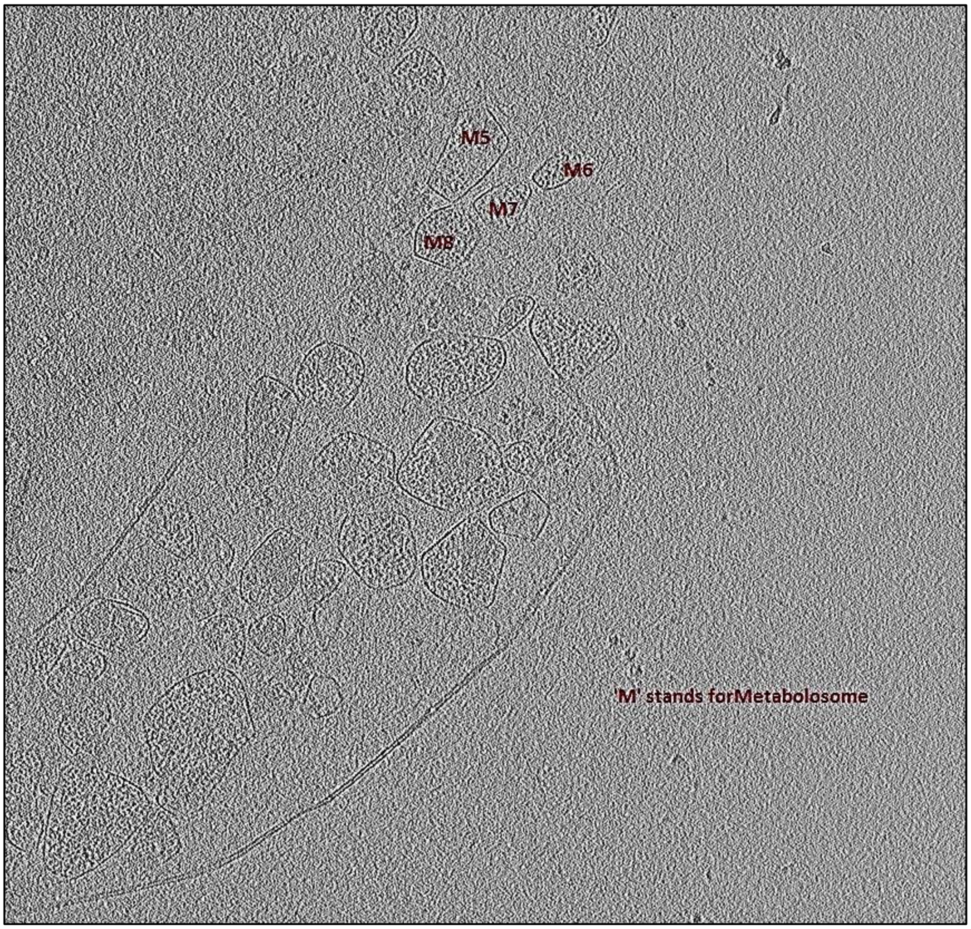

Tomogram 20 (View 3)

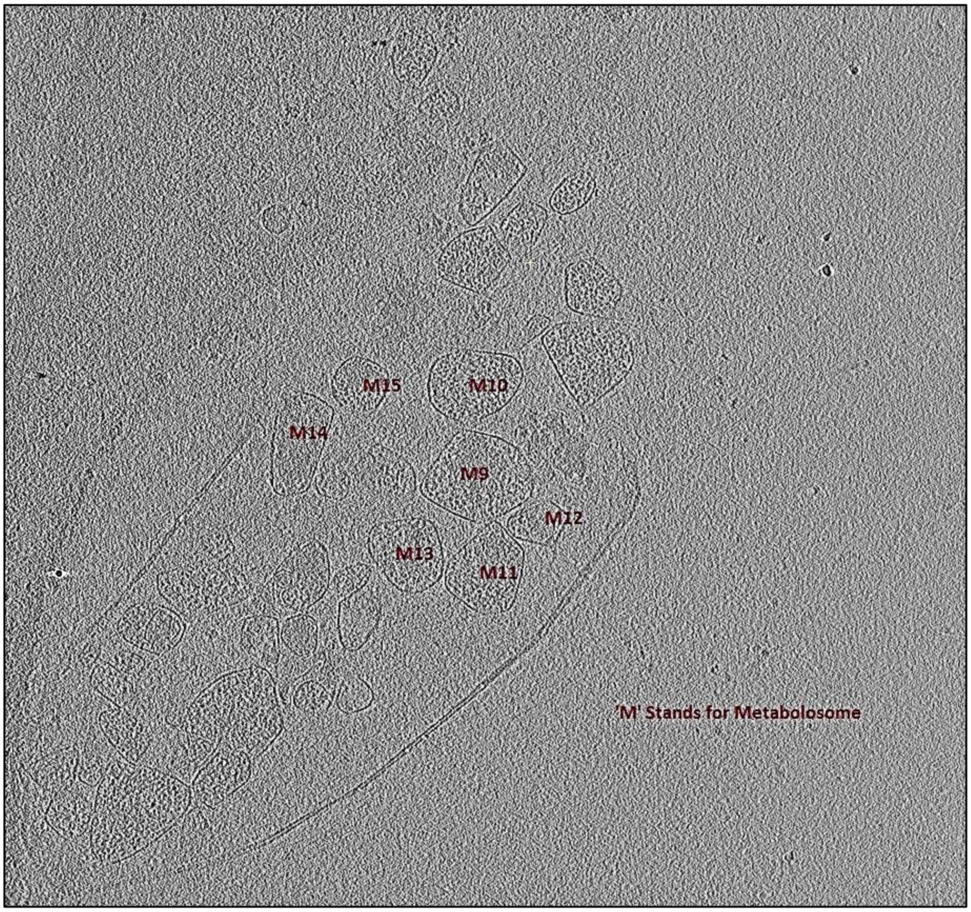

Tomogram 34 (View 1)

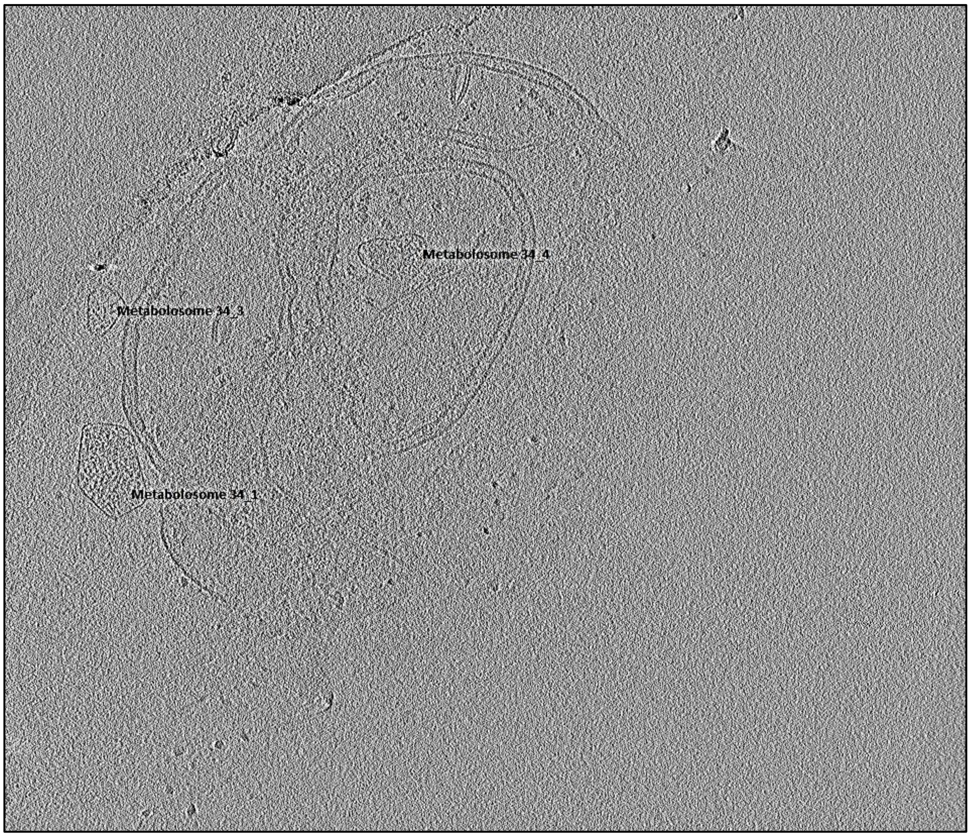

Tomogram 34 (View 2)

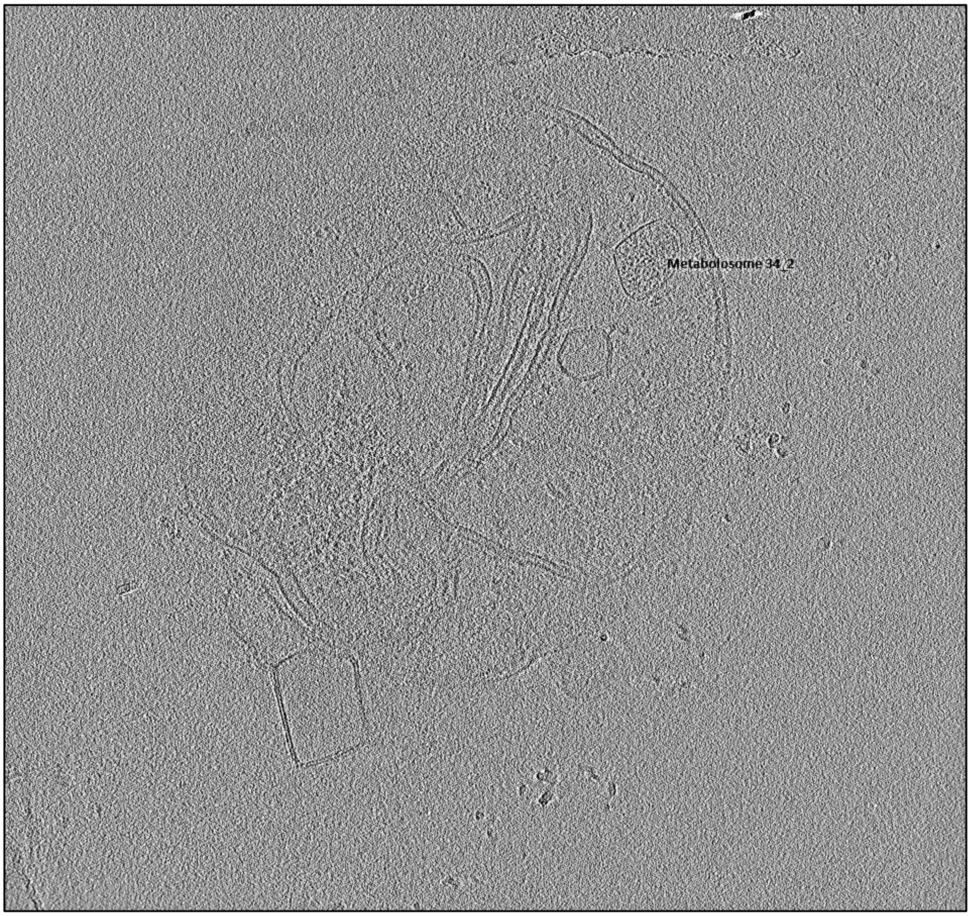

Tomogram 41

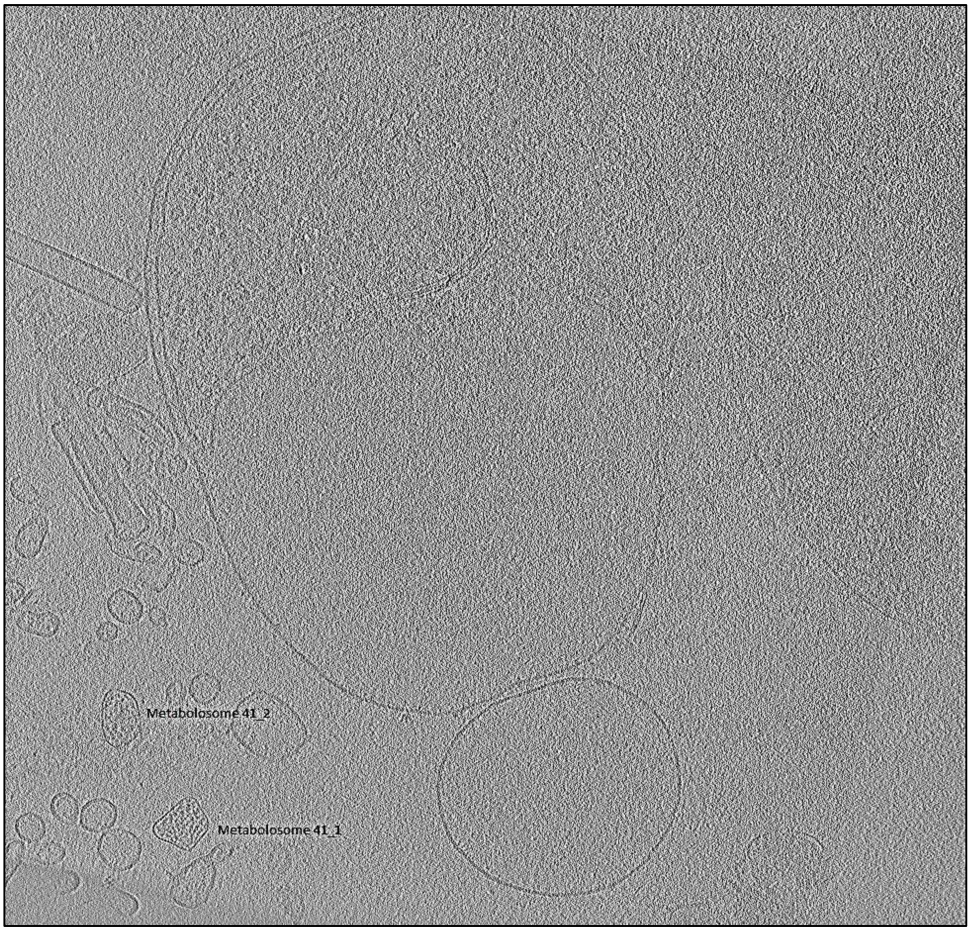

Supplement: Additional file 4: Figure S10. — (In separate file titled: 2D view of complete tomograms.pdf) Tomograms showing objects selected for reconstruction. (PDF 4567 kb) [file 12859_2016_1107_MOESM4_ESM.pdf]
