# Supplementary material for: Classification of polyhedral shapes from individual anisotropically resolved cryo-electron tomography reconstructions
Source: BMC Bioinformatics. 2016 Jun 13;17:234. doi: 10.1186/s12859-016-1107-5 (PMC4904361; doi:10.1186/s12859-016-1107-5)
Supplement: Additional file 5: Figure S11. — (In separate file titled: Individual BMC Shapes.pdf) 3-d volume renderings of individual reconstructed BMCs, followed by 3-d volume renderings of identified polyhedral shapes. (PDF 470 kb) [file 12859_2016_1107_MOESM5_ESM.pdf]

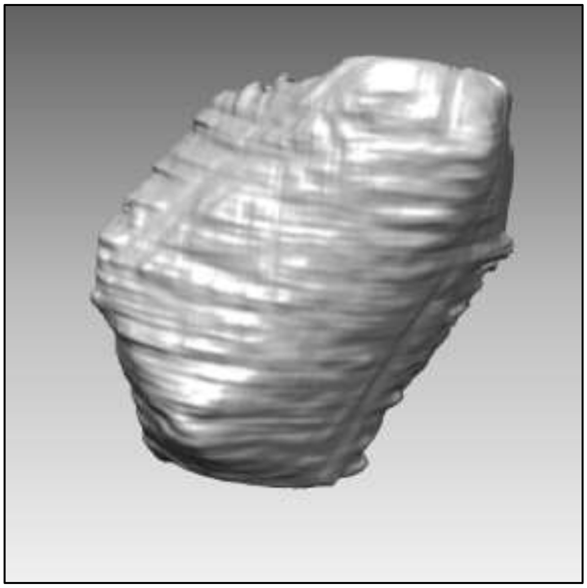
**Metabolosome 05_01**

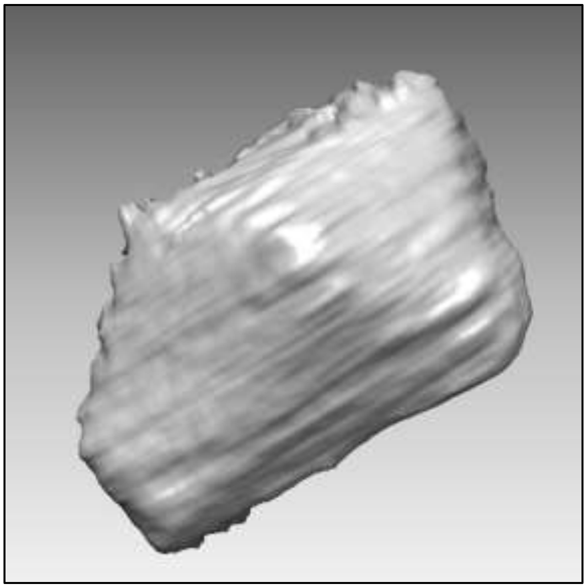
**Metabolosome 05_02**

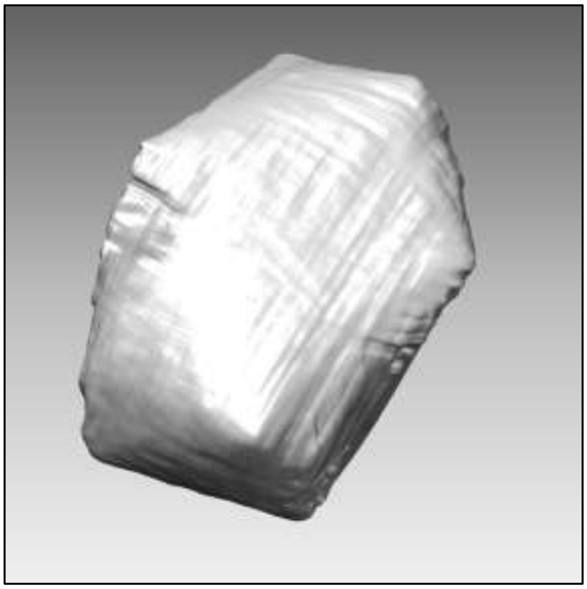
**Metabolosome 13_01**

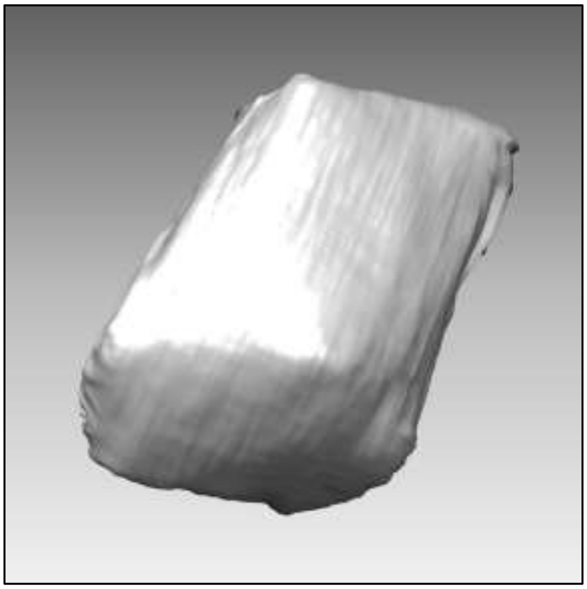
**Metabolosome 13_02**

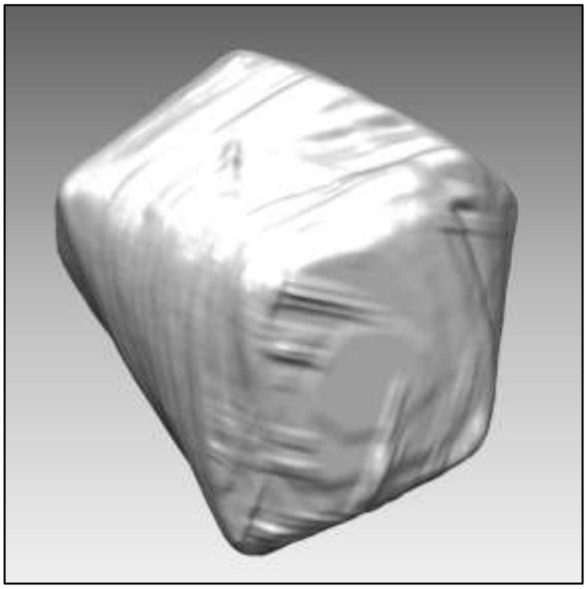
**Metabolosome 13_03**

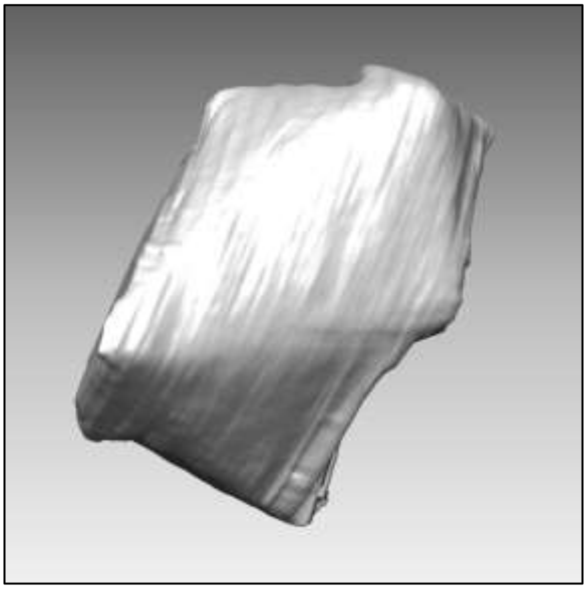
**Metabolosome 13_04**

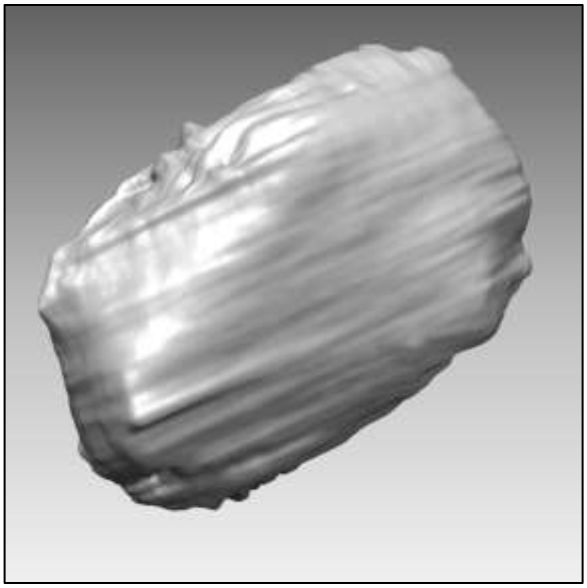
Metabolosome 13_05

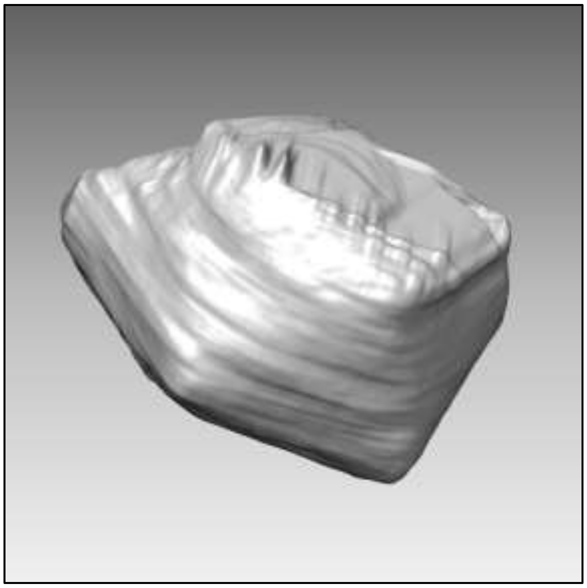
Metabolosome 13_06

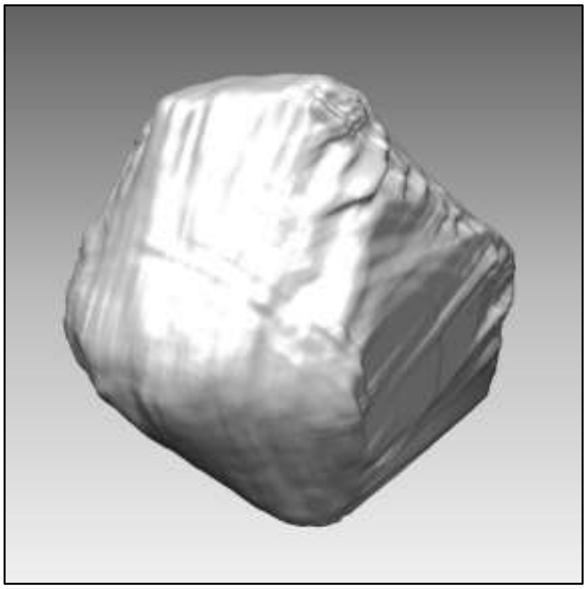
Metabolosome 13_07

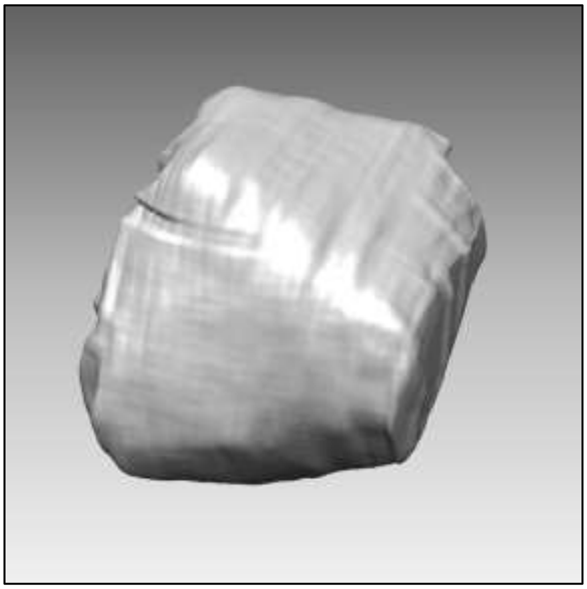
Metabolosome 13_08

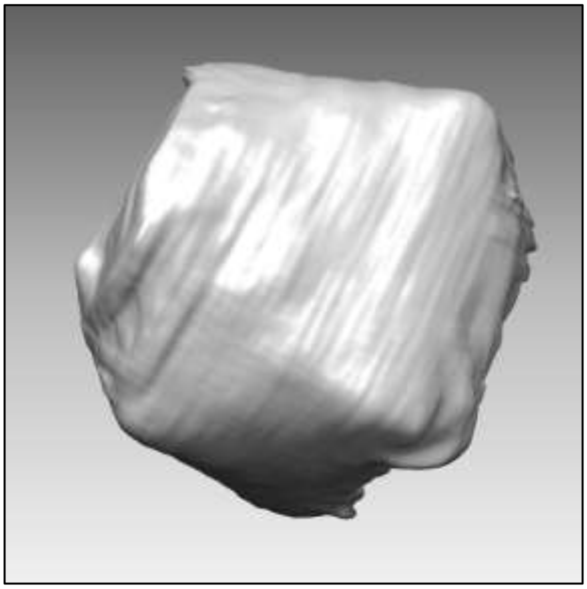
Metabolosome 13_09

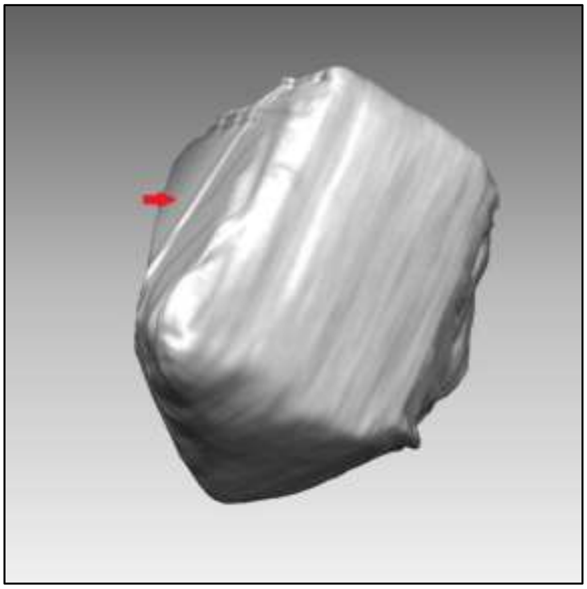
Metabolosome 13_12

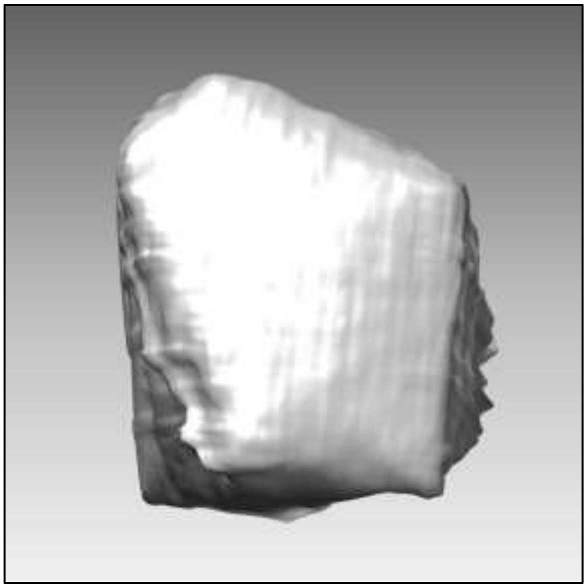
**Metabolosome 13_13**

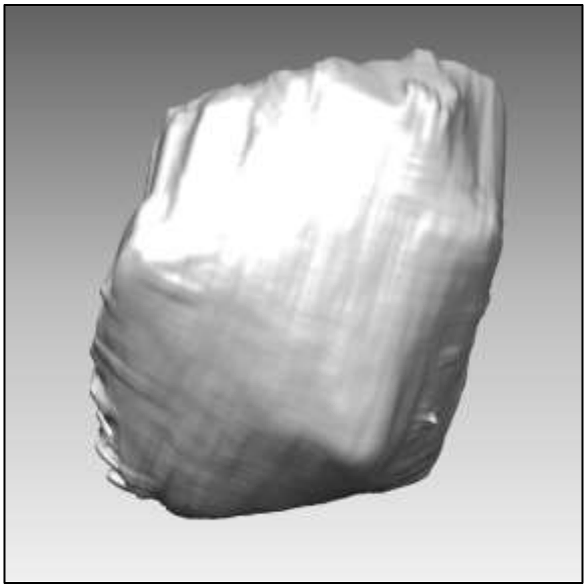
**Metabolosome 13_14**

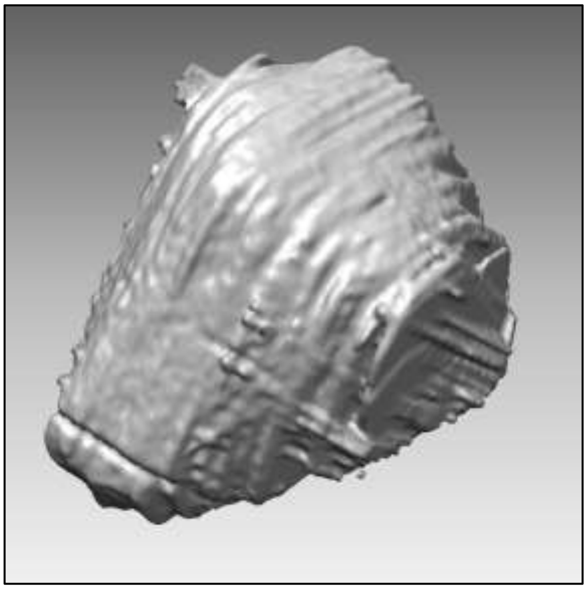
**Metabolosome 14_01**

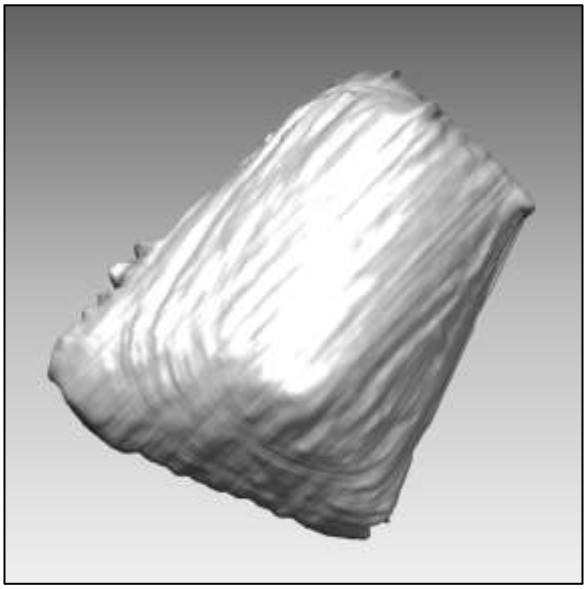
**Metabolosome 14_02**

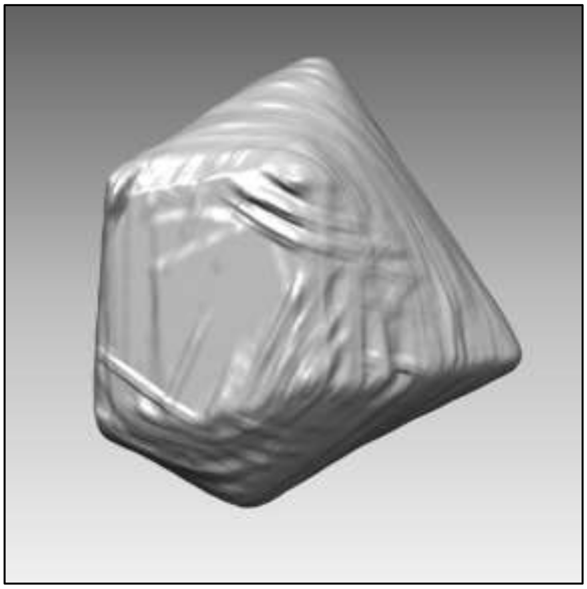
**Metabolosome 20_01**

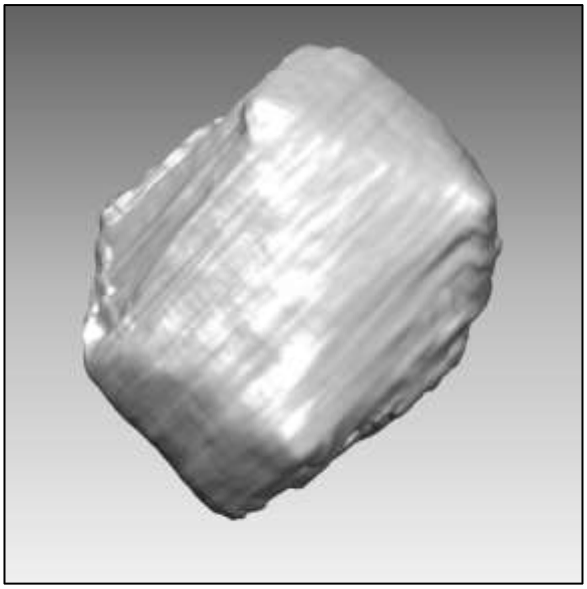
**Metabolosome 20_02**

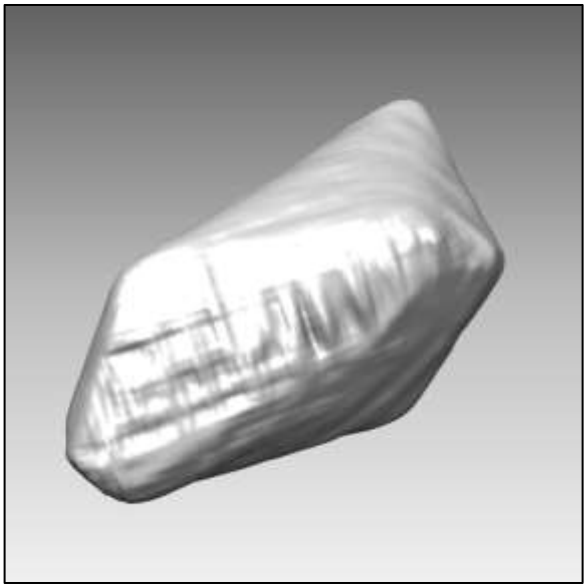

**Metabolosome 20_04**

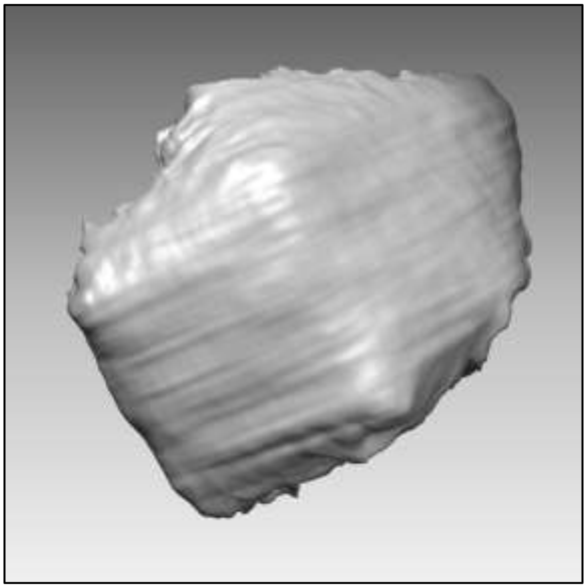

**Metabolosome 20_05**

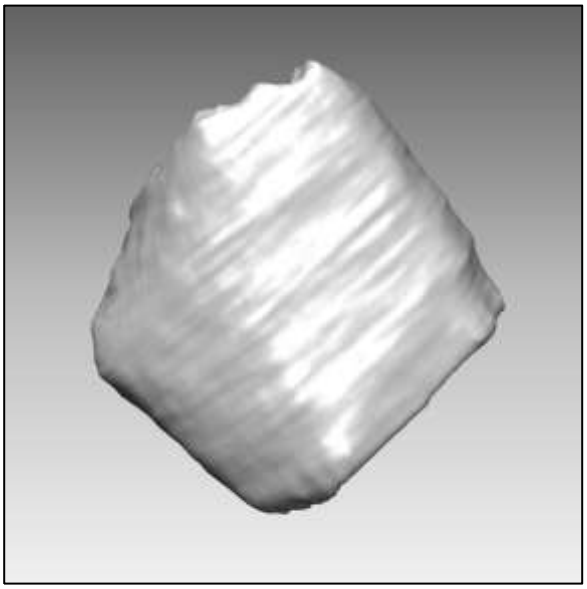

**Metabolosome 20_06**

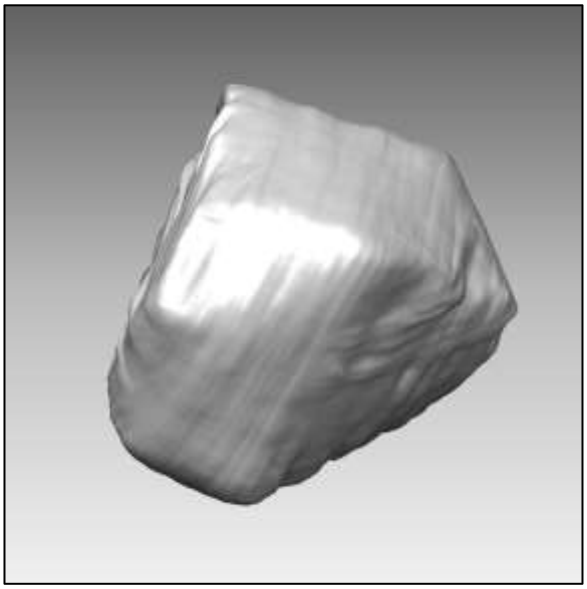

**Metabolosome 20_08**

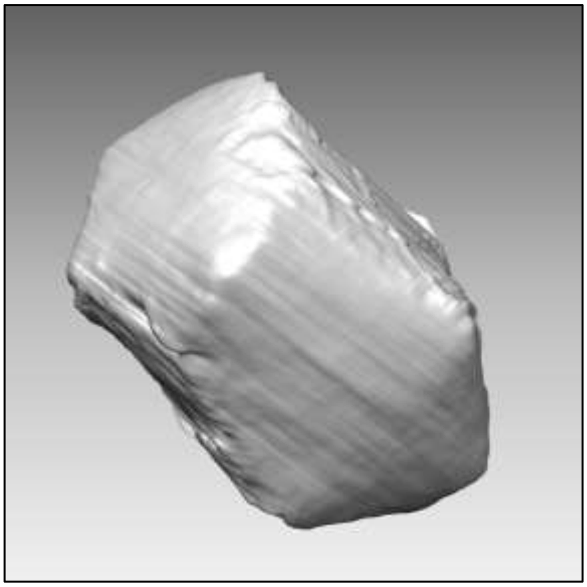

**Metabolosome 20_09**

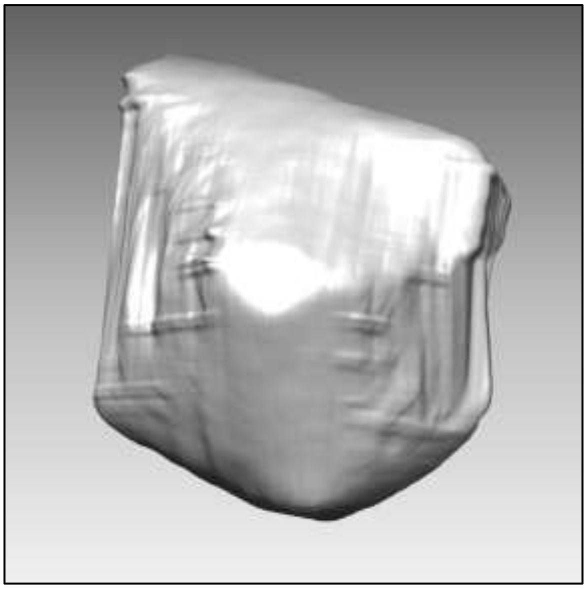

**Metabolosome 20_10**

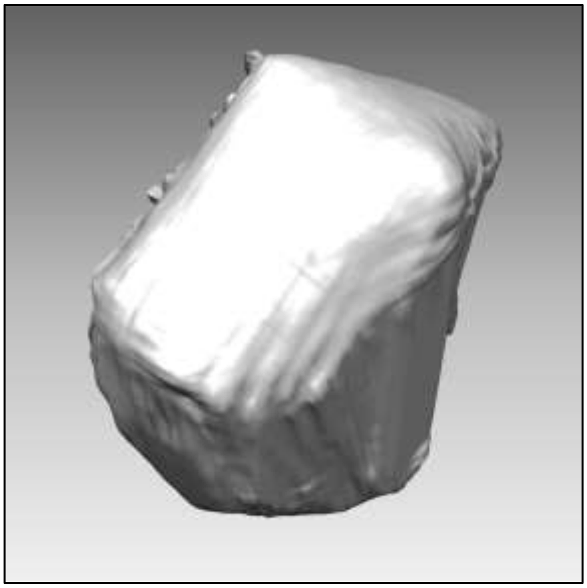
Metabolosome 20_11

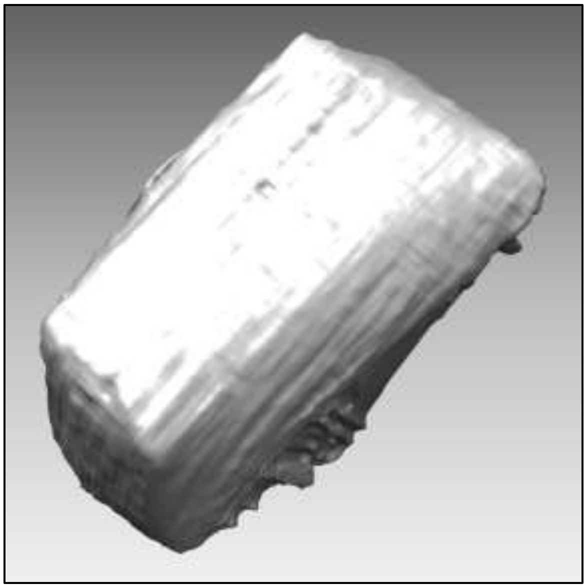
Metabolosome 34_01

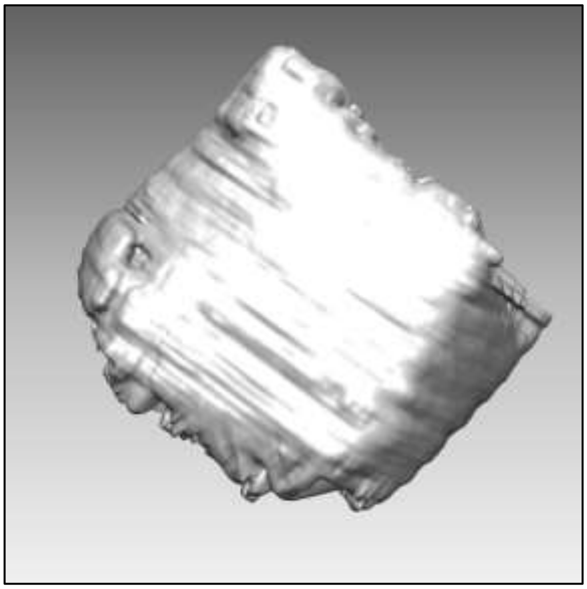
Metabolosome 34_02

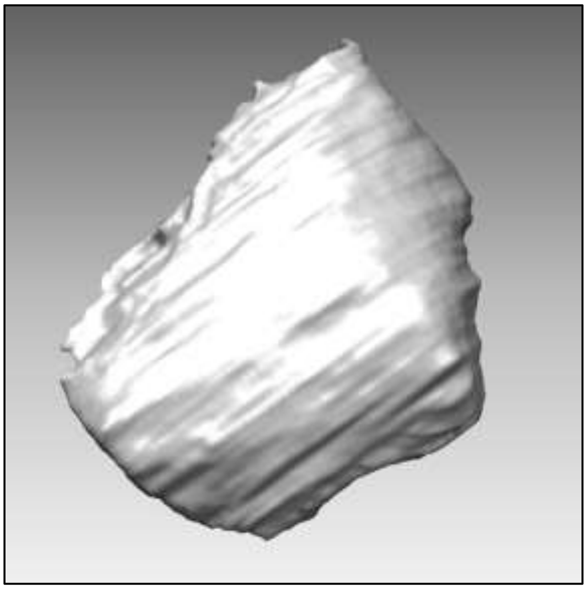
Metabolosome 34_03

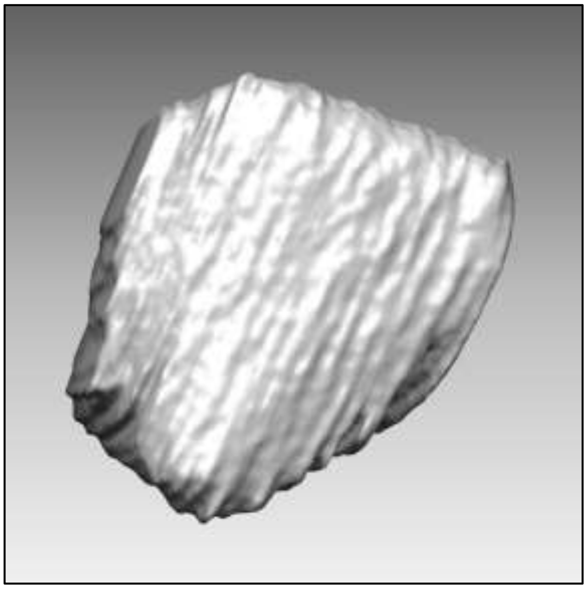
Metabolosome 41_01

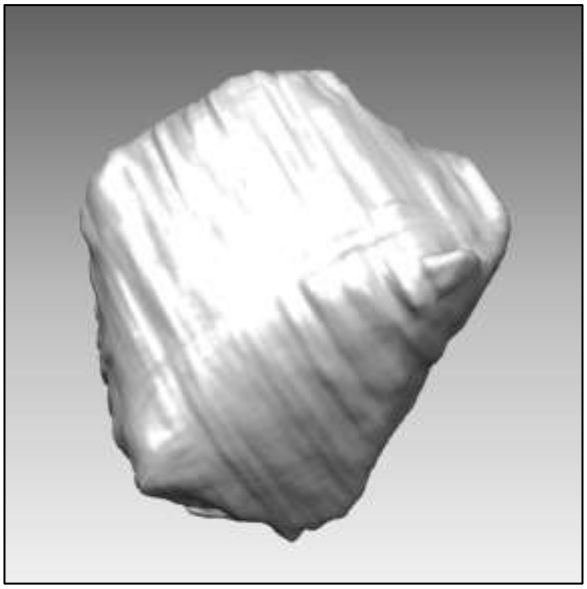
Metabolosome 41_02

The above images are 2D representations (screenshots) of 30 segmented metabolosomes. The images are visualized using Chimera UCSF. The captions include Metabolosome identifier number which helps to relate their individual shapes from the following table and figures.

| Metab. ID | Shape Name | Metab. ID | Shape Name |
|---|---|---|---|
| **05_01** | J17 - Gyroelongated square bipyramid | **14_02** | J86 - Sphenocorona |
| **05_02** | J11 - Gyroelongated pentagonal pyramid | **20_01** | J16 - Elongated pentagonal bipyramid |
| **13_01** | J16 - Elongated pentagonal bipyramid | **20_02** | J88 - Sphenomegacorona |
| **13_02** | J11 - Gyroelongated pentagonal pyramid | **20_04** | J50 - Biaugmented triangular prism |
| **13_03** | J16 - Elongated pentagonal bipyramid | **20_05** | J87 - Augmented sphenocorona |
| **13_04** | J86 - Sphenocorona | **20_06** | J86 - Sphenocorona |
| **13_05** | J86 - Sphenocorona | **20_08** | J87 - Augmented sphenocorona |
| **13_06** | J86 - Sphenocorona | **20_09** | J16 - Elongated pentagonal bipyramid |
| **13_07** | J16 - Elongated pentagonal bipyramid | **20_10** | J17 - Gyroelongated square bipyramid |
| **13_08** | J86 - Sphenocorona | **20_11** | J16 - Elongated pentagonal bipyramid |
| **13_09** | J88 - Sphenomegacorona | **34_01** | J16 - Elongated pentagonal bipyramid |
| **13_12** | J86 - Sphenocorona | **34_02** | J16 - Elongated pentagonal bipyramid |
| **13_13** | J54 - Augmented hexagonal prism | **34_03** | J16 - Elongated pentagonal bipyramid |
| **13_14** | J16 - Elongated pentagonal bipyramid | **41_01** | J62 - Metabidiminished icosahedron |
| **14_01** | J86 - Sphenocorona | **41_02** | J11 - Gyroelongated pentagonal pyramid |

The above table provides the metabolosomes shapes predicted by SVM. The letter 'J' stands for Johnson Solids and the following number is the Johnson solids serial number, followed by the solid names.

The following figures provide graphical representations of the predicted shapes of the metabolosomes. The images are from Wikipedia.

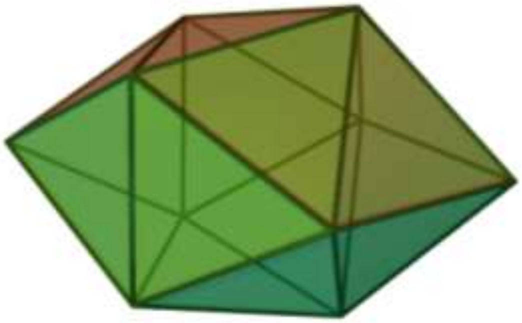

**05_01: J17 - Gyroelongated square bipyramid**

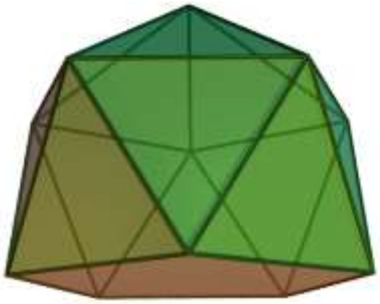

**05_02: J11 - Gyroelongated pentagonal pyramid**

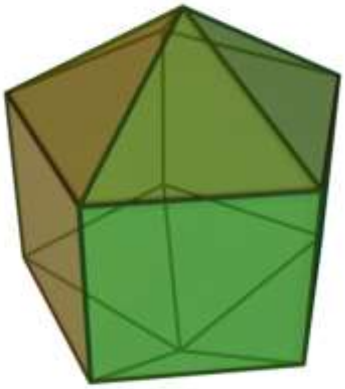

**13_01: J16 - Elongated pentagonal bipyramid**

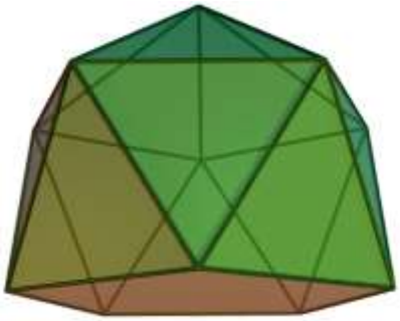

**13_02: J11 - Gyroelongated pentagonal pyramid**

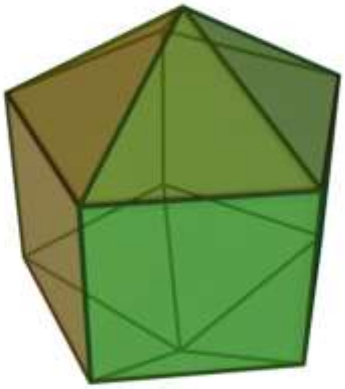

**13_03: J16 - Elongated pentagonal bipyramid**

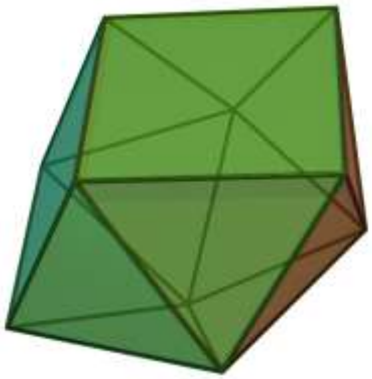

**13_04: J86 - Sphenocorona**

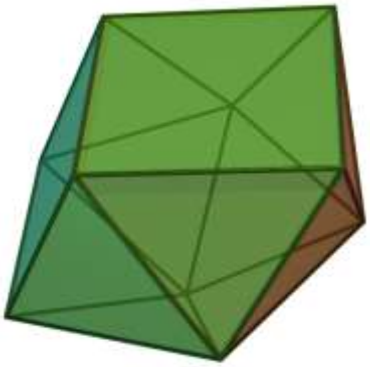

13_05: J86 - Sphenocorona

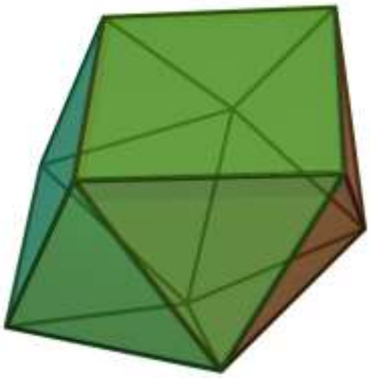

13_06: J86 - Sphenocorona

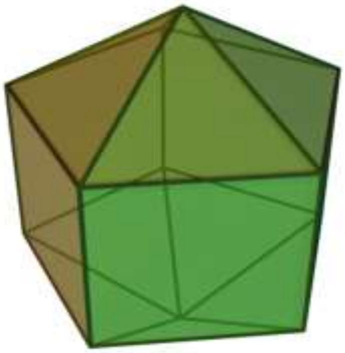

13_07: J16 - Elongated pentagonal bipyramid

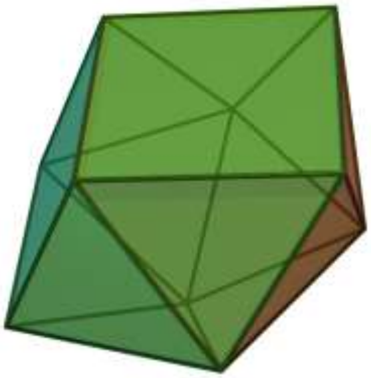

13_08: J86 - Sphenocorona

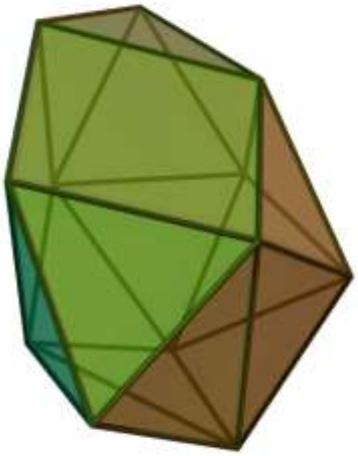

13_09: J88 - Sphenomegacorona

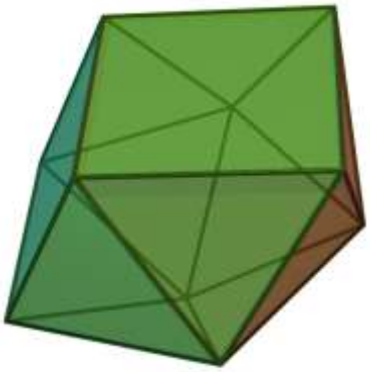

13_12: J86 - Sphenocorona

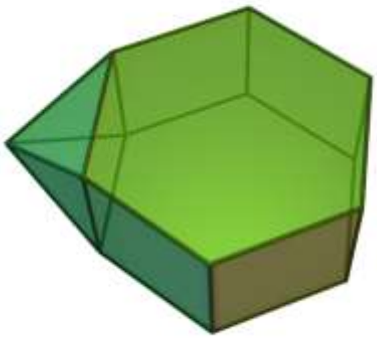

13_13: J54 - Augmented hexagonal prism

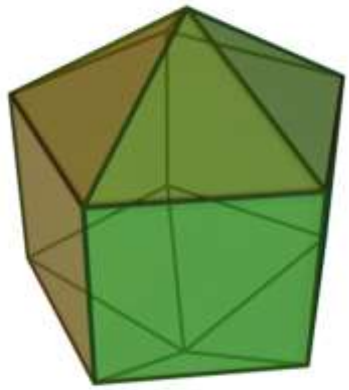

13_14: J16 - Elongated pentagonal bipyramid

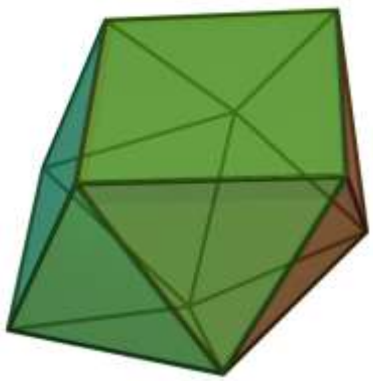

14_01: J86 - Sphenocorona

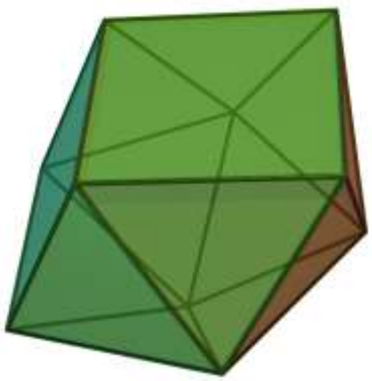

14_02: J86 - Sphenocorona

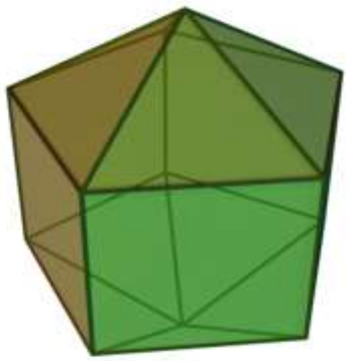

20_01: J16 - Elongated pentagonal bipyramid

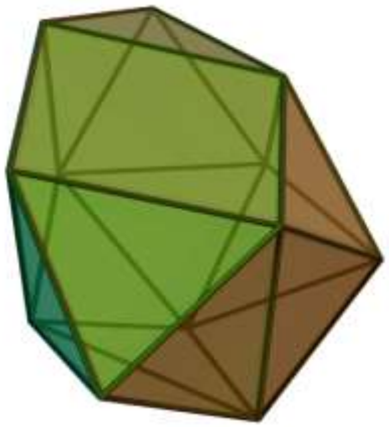

20_02: J88 - Sphenomegacorona

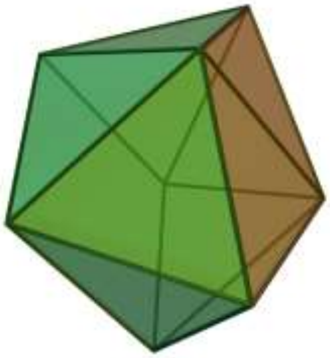

**20_04: J50 - Biaugmented triangular prism**

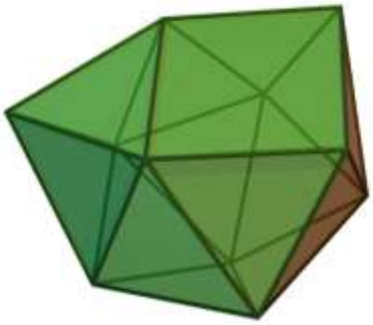

**20_05: J87 - Augmented sphenocorona**

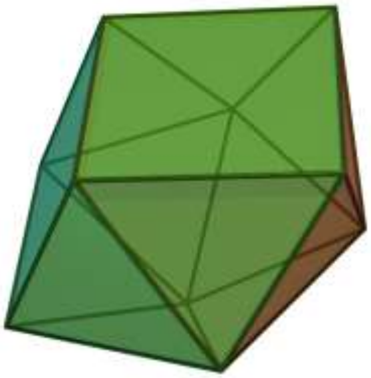

**20_06: J86 - Sphenocorona**

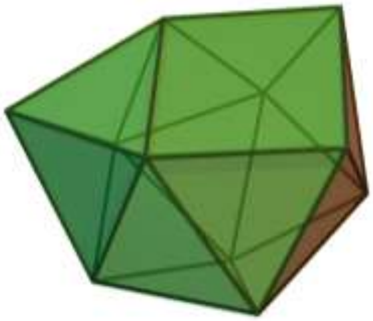

**20_08: J87 - Augmented sphenocorona**

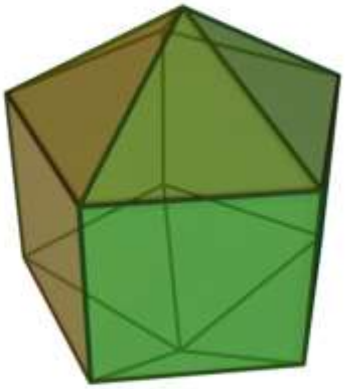

**20_09: J16 - Elongated pentagonal bipyramid**

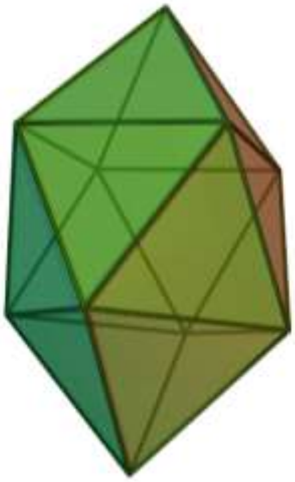

**20_10: J17 - Gyroelongated square bipyramid**

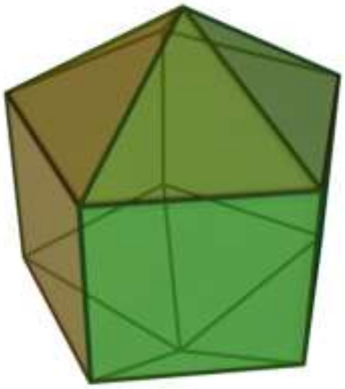

20_11: J16 - Elongated pentagonal bipyramid

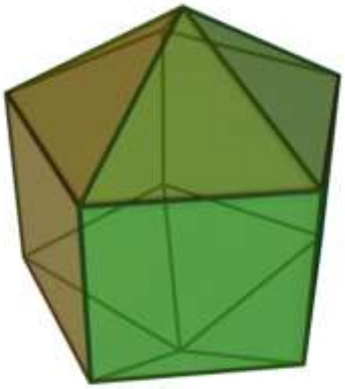

34_01: J16 - Elongated pentagonal bipyramid

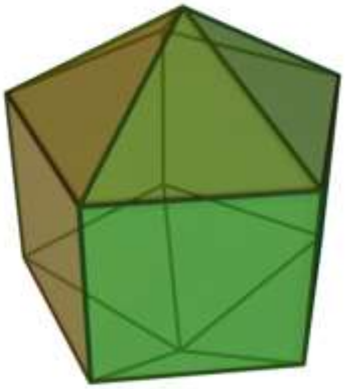

34_02: J16 - Elongated pentagonal bipyramid

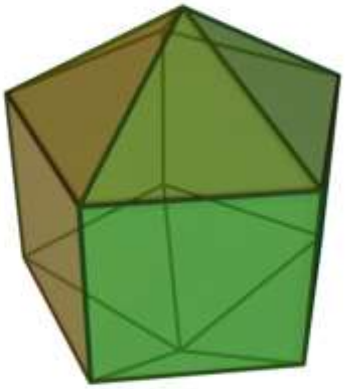

34_03: J16 - Elongated pentagonal bipyramid

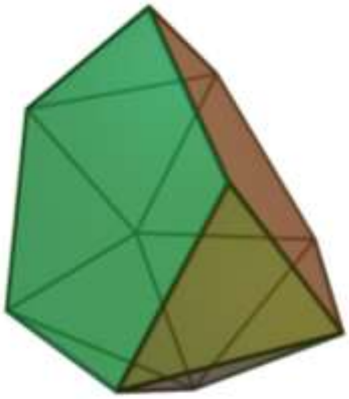

41_01: J62 - Metabidiminished icosahedron

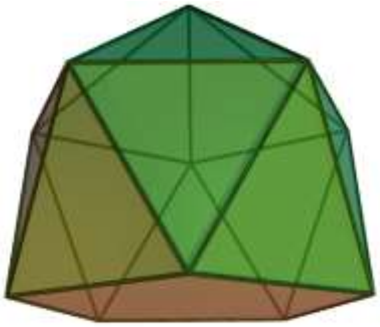

41_02: J11 - Gyroelongated pentagonal pyramid
